# Hospital-Course Infectious Complications Associated with In-Hospital Mortality in a Neurological Intensive Care Cohort: A Six-Year Retrospective Study

**DOI:** 10.3390/jcm15145562

**Published:** 2026-07-15

**Authors:** Simona Ioana Adriana Mlendea (Gălbineanu), Alin Kraft, Cristian Falup-Pecurariu, Tatiana Gianina Melicianu, Laurențiu Dănuț Nedelcu

**Affiliations:** 1 Department of Anaesthesia and Intensive Care, Brașov County Emergency Clinical Hospital, 500326 Brașov, Romania; sgalbineanu@gmail.com; 2 Doctoral School in Medicine, “Transilvania” University of Brașov, 500019 Brașov, Romania; laurnedelcu@yahoo.com; 3 Department of Medical-Surgical and Prophylactic Disciplines, Faculty of Medicine, Titu Maiorescu University of Bucharest, 031593 Bucharest, Romania; 4 Department of General Surgery, “General Doctor Aviator Victor Anastasiu” National Aeronautical and Space Medicine Institute, 010242 Bucharest, Romania; 5 Department of Neurology, Brașov County Emergency Clinical Hospital, 500326 Brașov, Romania; crisfp100@yahoo.com; 6 Faculty of Medicine, “Transilvania” University of Brașov, 500019 Brașov, Romania; 7 Department of Infectious Diseases, Brașov County Emergency Clinical Hospital, 500326 Brașov, Romania; dr.melicianutatiana@yahoo.com

**Keywords:** neurological intensive care unit, healthcare-associated infections, pneumonia, sepsis-related coding, urinary tract infection, in-hospital mortality, Glasgow Coma Scale, retrospective cohort

## Abstract

**Background/Objectives:** Infectious complications are frequent in neurological intensive care unit (ICU) patients and may contribute to in-hospital mortality. However, their independent association with in-hospital mortality in full neurological ICU cohorts remains insufficiently defined. This study evaluated documented hospital-course infectious complications as factors associated with in-hospital mortality in a six-year neurological ICU cohort. **Methods:** We performed a retrospective, single-center cohort study including all available neurological ICU admission episodes recorded between 1 January 2020 and 31 December 2025. The primary outcome was in-hospital mortality. Infectious variables included pneumonia, COVID-related pneumonia, urinary tract infection, pressure sore or pressure sore-related infection, sepsis-related coding, and any infectious complication. Multivariable logistic regression was used to assess independent associations with mortality. The primary model included individual infectious complications without Glasgow Coma Scale (GCS), while a GCS-adjusted model was used as a sensitivity analysis. Incremental model analysis, model validation/calibration, and COVID-related sensitivity analyses were also performed. **Results:** The cohort included 5509 neurological ICU admission episodes; 999 ended in in-hospital death, corresponding to a mortality rate of 18.1%. Any infectious complication was documented in 1911 episodes (34.7%). Pneumonia was the most frequent infectious complication (22.2%) and remained independently associated with mortality in the primary model (adjusted OR 6.82, 95% CI 5.70–8.18; *p* < 0.001) and in the GCS-adjusted model (adjusted OR 5.25, 95% CI 4.05–6.80; *p* < 0.001). Sepsis-related coding, interpreted as a documentation-based marker of severe systemic infectious deterioration rather than formally adjudicated sepsis, showed the strongest adjusted association with death (adjusted OR 12.40, 95% CI 6.53–23.54; *p* < 0.001). Urinary tract infection and pressure sore-related infection were associated with mortality in unadjusted analyses but not after adjustment. **Conclusions:** Pneumonia and sepsis-related coding were robustly and independently associated with in-hospital mortality. Infectious complications added mortality-related information beyond baseline clinical variables and should be integrated into neurological ICU risk assessment and infection-surveillance strategies.

## 1. Introduction

Healthcare-associated infections remain a major challenge in intensive care medicine, but their relevance is particularly pronounced in neurological intensive care populations. Patients admitted to neurological intensive care units frequently present with severe acute brain injury, impaired consciousness, dysphagia, reduced cough and airway protection, prolonged immobilization, autonomic and immune dysregulation, and exposure to invasive devices. These factors create a clinical context in which systemic infections are both common and difficult to diagnose, especially because fever, leukocytosis, altered mental status, respiratory deterioration, and inflammatory responses may overlap with the neurological disease process itself [1,2]. Previous neurocritical care surveillance data have shown that pneumonia, urinary tract infection, and device-associated infections are among the most frequent healthcare-associated infections in neurological ICUs, and that these complications contribute substantially to length of stay and resource utilization [3]. At the European level, intensive care units continue to represent high-risk environments for healthcare-associated infections, with pneumonia, bloodstream infection, and urinary tract infection forming the principal infection categories under surveillance [4]. Large international ICU data have similarly shown that infection is highly prevalent among critically ill patients and is independently associated with hospital mortality [5]. In severe neurological ICU populations, in-hospital mortality reflects the combined effect of the primary neurological insult, neurological severity, comorbidity burden, physiological instability, and secondary complications acquired during hospitalization. Among these complications, infections are particularly relevant because they are potentially modifiable and may provide additional mortality-related information beyond baseline neurological status [1,3,6].

Among neurological patients, stroke-associated infections have received substantial attention. Previous systematic reviews and meta-analyses have shown that infections complicate a relevant proportion of acute stroke cases, with pneumonia and urinary tract infection representing the most frequent phenotypes and pneumonia showing the strongest association with adverse outcomes [6,7]. These data support the importance of infection prevention, early recognition, and accurate documentation in neurological and stroke care.

Pneumonia is particularly relevant in neurocritical care because it lies at the intersection between impaired consciousness, dysphagia, reduced airway protection, aspiration risk, mechanical ventilation exposure, immobility, and systemic inflammatory response. Consensus recommendations for stroke-associated pneumonia have emphasized the need for standardized terminology, diagnostic criteria, and careful antibiotic decision-making, reflecting the diagnostic uncertainty and clinical heterogeneity of this complication [8,9]. Recent evidence further supports the association between stroke-associated pneumonia and increased mortality or poor functional outcomes [10].

Other infectious or immobility-related complications, including urinary tract infection and pressure sore or pressure sore-related infection, are also clinically relevant in neurological ICU populations. Urinary tract infection may be favored by bladder dysfunction, urinary catheter exposure, impaired mobility, older age, and comorbidities, while pressure sore-related complications reflect prolonged immobility, critical illness, nutritional vulnerability, and care dependency [11,12]. Although these complications may be less strongly associated with mortality than pneumonia or systemic infectious deterioration, they remain important markers of hospital-course complexity and quality-of-care priorities.

Sepsis-related events represent an additional high-risk domain in critically ill patients. In retrospective administrative or clinical databases, however, sepsis may be difficult to adjudicate formally unless standardized organ dysfunction criteria, timing, microbiology, and source-control data are consistently available. For this reason, sepsis-related documentation or coding should be interpreted cautiously, particularly when derived from routine retrospective datasets. Nevertheless, the presence of sepsis-related coding may identify a subgroup of patients with severe systemic infectious deterioration and high mortality risk.

Despite the broad international literature on post-stroke infections and neuro-ICU healthcare-associated infections, several gaps remain. Many studies focus on specific diagnostic groups, such as ischemic stroke, hemorrhagic stroke, or neurosurgical populations; others report surveillance incidence rates without directly modeling in-hospital mortality across the full neurological ICU population. In addition, pneumonia and urinary tract infection have often been considered together as post-stroke infection outcomes, while fewer studies have evaluated individual infectious complications simultaneously in adjusted mortality models. From a methodological perspective, infectious complications occurring during hospitalization are particularly challenging to interpret because they may function both as markers of baseline severity and as hospital-course complications. This makes multivariable adjustment, sensitivity analysis for neurological severity, and careful avoidance of inappropriate time-to-event assumptions essential.

Romanian data on healthcare-associated infections have specific relevance because national surveillance and reporting frameworks have evolved substantially, while under-reporting, infrastructure limitations, and implementation barriers remain recognized challenges [13]. In the neurological intensive care setting, Romanian evidence remains limited. A recent retrospective descriptive study from a Romanian neurological ICU described the burden of documented healthcare-associated infections among deceased stroke patients, with pneumonia representing the dominant infectious complication; however, by design, that study was restricted to fatal cases and could not compare mortality-outcome groups or evaluate infectious complications as factors associated with mortality in a full cohort [14].

The present study was therefore designed to address this gap by analyzing all available neurological ICU admission episodes recorded over a six-year period, including both survivors and admissions ending in in-hospital death. The originality of the study resides in its full-cohort neurological ICU design, its survivor-versus-non-survivor analytical framework, and its focus on individual infectious complications as factors associated with in-hospital mortality. The study also evaluates whether infectious complications add mortality-related information beyond baseline demographic, neurological, and comorbidity variables, and whether the association between pneumonia and mortality is robust after sensitivity analyses including neurological severity and COVID-related pneumonia.

The aim of this study was to evaluate infectious complications as factors associated with in-hospital mortality in a six-year retrospective neurological intensive care cohort. The specific objectives were to describe the burden of documented infectious complications, compare baseline and infectious profiles according to survival status, assess the unadjusted and adjusted associations between individual infectious complications and in-hospital mortality, evaluate the incremental mortality-related contribution of infections beyond clinical variables, and test the robustness of the main findings in Glasgow Coma Scale-adjusted and COVID-related sensitivity analyses.

## 2. Materials and Methods

### 2.1. Study Design and Setting

This study was designed as a retrospective, single-center cohort study evaluating infectious complications as factors associated with in-hospital mortality among patients admitted to a neurological intensive care unit over a six-year period. The study was based on routinely collected institutional clinical data from neurological intensive care practice.

The study was conducted in a neurological intensive care unit caring primarily for patients with acute neurological conditions requiring intensive monitoring or treatment, including ischemic stroke, hemorrhagic stroke, transient ischemic attack, stroke sequelae, and other severe neurological disorders. The unit was not designed as a primary postoperative surgical intensive care unit. Respiratory support and other intensive care interventions were provided according to institutional clinical practice when required; however, detailed variables on intubation, duration of mechanical ventilation, tracheostomy, central venous catheter exposure, urinary catheter duration, vasopressor use, and nutritional support were not uniformly available in the retrospective dataset.

The analytical design was conceived as a full-cohort analysis of neurological intensive care admission episodes, including both survivor admissions and admissions ending in in-hospital death. This approach was selected to allow direct comparison according to in-hospital mortality status and to assess whether documented infectious complications provided mortality-related information beyond demographic characteristics, neurological diagnosis, and available comorbidities.

### 2.2. Study Population and Observation Period

The study population consisted of all available admission episodes recorded in the neurological intensive care unit between 1 January 2020 and 31 December 2025.

The unit of analysis was predefined as the neurological intensive care admission episode. Following reviewer comments, the database was manually rechecked for duplicate or recurrent patient entries. No duplicated patients or recurrent admissions of the same patient were identified. Therefore, each row in the final analytical dataset represented one distinct neurological ICU admission corresponding to one individual patient. Identical duplicate records were also assessed during data cleaning, and no admission episodes were excluded from the final analytical cohort. The final analytical cohort included 5509 neurological intensive care admissions.

### 2.3. Data Sources and Database Preparation

The source dataset consisted of six annual databases corresponding to the years 2020-2025. These annual files were inspected, harmonized, and merged into a unified working database. The annual databases were derived from routinely collected institutional neurological ICU records and clinical documentation. Infectious complications were identified from documented entries available in the source datasets and related clinical documentation fields. The study did not use prospective infection adjudication, and systematic re-evaluation of all full medical charts according to standardized surveillance definitions was not feasible retrospectively. Therefore, the infection variables should be interpreted as documented infectious complications recorded during routine clinical care rather than prospectively adjudicated infection endpoints. The merged database was subsequently cleaned, deidentified, and structured for statistical analysis.

Direct personal identifiers, including patient name and institutional file number, were reviewed only during the data-cleaning and duplicate-checking stage and were removed before creation of the final anonymized analytical dataset. Following manual verification, no duplicated patients or recurrent admissions of the same patient were identified. A new anonymized study identifier was generated for each record. For technical traceability, the final database retained the source year and source row number, without preserving direct patient identifiers.

Database preparation included harmonization of column names, standardization of categorical variables, binary recoding of clinical variables, correction of clearly identifiable data-entry errors, and documentation of all relevant recoding decisions. Calendar years recorded as 3021 and 3024 were corrected to 2021 and 2024, respectively, because these were considered obvious typographical errors. These corrections were documented in the data-cleaning audit log.

No imputation was performed. Descriptive analyses were conducted using available data for each variable. Multivariable models were fitted as complete-case analyses according to the availability of all covariates included in each model.

### 2.4. Outcome Definition

The primary outcome was in-hospital mortality. A binary variable was created and coded as 1 for admission episodes ending in in-hospital death and 0 for episodes in which the patient survived to discharge or had no documented in-hospital death.

This variable served as the dependent variable in the univariable and multivariable logistic regression analyses.

### 2.5. Infectious Complication Variables

The main exposure domain consisted of documented infectious complications recorded during the index hospitalization. These variables were considered hospital-course variables rather than baseline exposures. The following binary variables were created: pneumonia, COVID-related pneumonia, urinary tract infection, pressure sore or pressure sore-related infection, sepsis-related coding, and any infectious complication.

Pneumonia was coded as present when the source database contained pneumonia-related documentation. COVID-related pneumonia was retained as a separate category when COVID-related wording was documented. Urinary tract infection was coded as present when urinary infection documentation or pathogen-specific urinary infection wording was identified. Pressure sore or pressure sore-related infection was coded as present when pressure sore, escara, infected pressure ulcer, pressure sore-related infection, or equivalent wording was documented. Because standardized pressure injury staging, microbiological confirmation, and formal wound infection criteria were not uniformly available, this variable was analyzed as documentation of pressure sore or pressure sore-related infection rather than as a prospectively adjudicated infected pressure injury endpoint.

Sepsis-related coding was assigned when the source dataset contained sepsis-related wording, including documented sepsis or septic shock where present. Uniform ICD-based adjudication and complete Sepsis-3 reclassification were not available in the retrospective dataset. Therefore, this variable was interpreted as a documentation-based marker of severe systemic infectious deterioration rather than as formally adjudicated Sepsis-3 sepsis or septic shock.

A composite variable, any infectious complication, was created and coded as present when at least one of the main documented infectious complication variables was positive. COVID-related pneumonia was retained for descriptive and sensitivity analyses. It was not entered as a separate covariate in the primary individual-infection model, in which pneumonia was treated as the main pneumonia variable. Dedicated COVID-related sensitivity analyses were subsequently performed to evaluate whether the association between pneumonia and in-hospital mortality was driven primarily by COVID-related pneumonia.

### 2.6. Neurological Diagnosis and Comorbidity Variables

Neurological diagnosis variables were derived from the documented source fields and included ischemic stroke, hemorrhagic stroke, transient ischemic attack, and stroke sequelae. These variables were retained as binary indicators. For regression modeling, hemorrhagic stroke was evaluated in relation to ischemic stroke where appropriate, given its clinical relevance and observed association with mortality.

Comorbidity variables were harmonized and coded as binary indicators. These included hypertension, ischemic heart disease and/or previous myocardial infarction, atrial fibrillation, diabetes mellitus, obesity, renal disease, hepatic disease, coagulopathy, previous stroke, malignancy, and anticoagulation. For binary clinical and comorbidity fields structured as presence/absence documentation, negative, absent, or blank entries were coded as 0 when the source format supported this interpretation. Some comorbidity variables, including obesity, reflected routine clinical documentation rather than systematic prospective adjudication and were therefore interpreted as recorded comorbidity documentation variables. Ambiguous entries were reviewed during data cleaning and documented when relevant.

### 2.7. Severity and Physiological Variables

Available continuous variables included age, initial Glasgow Coma Scale score, initial systolic blood pressure, initial diastolic blood pressure, and length of stay. Initial Glasgow Coma Scale score was considered an important marker of neurological severity. However, because it was available in only approximately 54.6% of the full cohort, it was not included in the primary multivariable model. Instead, a separate Glasgow Coma Scale-adjusted sensitivity model was constructed.

Initial blood pressure values were described in the cohort and compared according to in-hospital mortality status. Length of stay was summarized descriptively but was not included as a primary explanatory variable for mortality because it is influenced by both discharge timing and early death.

### 2.8. Statistical Analysis

Continuous variables were summarized using median and interquartile range. Mean and standard deviation were also reported where useful for descriptive purposes. Categorical variables were summarized as absolute frequencies and percentages.

Comparisons between survivor admissions and admissions ending in in-hospital death were performed using appropriate tests according to variable type and distribution. Continuous variables were compared using non-parametric methods when distributional assumptions were not met. Categorical variables were compared using chi-square or equivalent tests, as appropriate. These comparisons were considered descriptive and exploratory.

Annual distributions of admissions, mortality, and infectious complications were reported descriptively, and no formal temporal trend model was used. Unadjusted odds ratios with 95% confidence intervals were calculated for categorical factors associated with in-hospital mortality.

Multivariable logistic regression was used to identify factors independently associated with in-hospital mortality. The primary model included individual documented infectious complications as hospital-course variables, without Glasgow Coma Scale score. This model was selected as the main analytical model because it preserved almost the full cohort and allowed separate assessment of pneumonia, urinary tract infection, pressure sore or pressure sore-related infection, and sepsis-related coding. Covariates included age, sex, calendar year, major neurological diagnosis variables, and clinically relevant comorbidities.

A Glasgow Coma Scale-adjusted sensitivity model was constructed by adding initial Glasgow Coma Scale score to the individual-infection model. This model was used to evaluate whether the associations between infectious complications and in-hospital mortality persisted after adjustment for neurological severity. Because this model was restricted to admissions with available Glasgow Coma Scale data and complete covariate information, it was interpreted as a sensitivity analysis rather than as the primary model.

All multivariable logistic regression analyses were performed as complete-case analyses according to the covariates included in each model. Variable-level completeness was reported in the Supplementary Material. Multiple imputation was not performed because missingness was considered likely to reflect routine documentation practices and clinical workflow rather than a clearly missing-at-random mechanism, particularly for GCS and original detail fields. In addition, the main infection variables were documentation-derived hospital-course variables rather than prospectively adjudicated baseline covariates. Therefore, complete-case analyses were retained, complemented by explicit reporting of variable completeness and comparison of included versus excluded observations for the main multivariable models.

Model results were reported as adjusted odds ratios with 95% confidence intervals and *p* values. A two-sided *p* value < 0.05 was considered statistically significant. Data preparation, cleaning, and tabulation were performed using Microsoft Excel (Microsoft Corp., Redmond, WA, USA). Statistical analyses were performed using IBM SPSS Statistics version 26 (IBM Corp., Armonk, NY, USA), with additional spreadsheet-based calculations used where appropriate.

### 2.9. Incremental Model Analysis

To evaluate whether infectious complications added mortality-related information beyond baseline clinical variables, an incremental model analysis was performed. A clinical-only logistic regression model including demographic variables, calendar year, neurological diagnosis variables, and comorbidities was compared with a clinical-plus-infections model that additionally included pneumonia, urinary tract infection, pressure sore or pressure sore-related infection, and sepsis-related coding.

Model performance was compared using the area under the receiver operating characteristic curve, McFadden pseudo-R^2^, Akaike information criterion, Bayesian information criterion, and likelihood-ratio testing. This incremental analysis was performed both in the primary cohort without Glasgow Coma Scale score and in the Glasgow Coma Scale-available subset as a sensitivity analysis.

### 2.10. Model Validation and Calibration

Model discrimination was evaluated using the area under the receiver operating characteristic curve. Predictive accuracy was assessed using the Brier score and log loss. Calibration was evaluated using the calibration intercept, calibration slope, decile-based calibration summaries, the Hosmer–Lemeshow goodness-of-fit test, and calibration curves based on observed versus predicted mortality risk.

Internal validation was performed using bootstrap resampling. Optimism-corrected estimates were calculated for model discrimination and calibration, including bootstrap-corrected area under the receiver operating characteristic curve, Brier score, and calibration slope. This validation approach was applied to both the primary model and the Glasgow Coma Scale-adjusted sensitivity model.

Multicollinearity was assessed using variance inflation factors and tolerance values for both the primary no-GCS model and the GCS-adjusted sensitivity model. Calibration curves and multicollinearity results were provided in the Supplementary Material.

### 2.11. Decision Curve Analysis

Decision curve analysis was performed as an exploratory assessment of the potential net benefit of adding documented infectious complications to clinical-only models. Net benefit was calculated across a range of threshold probabilities and compared between clinical-only models and clinical-plus-infections models. This analysis was performed for both the primary no-GCS cohort and the GCS-available sensitivity cohort. The results were interpreted in the context of the retrospective design and the hospital-course nature of infectious complications.

### 2.12. COVID-Related Sensitivity Analysis

Because the study period included the COVID-19 pandemic and pneumonia was the main infectious complication of interest, additional sensitivity analyses were performed to assess whether the association between pneumonia and in-hospital mortality was driven primarily by COVID-related pneumonia.

First, the primary multivariable logistic regression model was repeated after excluding cases with COVID-related pneumonia. Second, pneumonia was separated into non-COVID pneumonia and COVID-related pneumonia within the full-cohort model. These analyses were also repeated in the Glasgow Coma Scale-available subset.

The purpose of these analyses was to determine whether non-COVID pneumonia remained independently associated with in-hospital mortality after accounting for COVID-related pneumonia.

### 2.13. Rationale for Not Using Cox Regression or Kaplan–Meier Analysis as Primary Analyses

Time-to-event analyses, including Cox proportional hazards regression and Kaplan–Meier curves, were not used as primary inferential analyses for infectious complications. Although length-of-stay and survival-time variables were available, the exact dates of onset of pneumonia, urinary tract infection, pressure sore-related infection, and sepsis-related coding were not consistently available in the source database.

Because infectious complications were hospital-course variables rather than baseline exposures, treating them as fixed covariates from admission would create a risk of immortal time bias. Patients must survive long enough and remain hospitalized long enough for such complications to be documented. Therefore, logistic regression for in-hospital mortality was considered the most appropriate primary analytical framework for the available data. Accordingly, the resulting estimates were interpreted as associations between documented hospital-course infectious complications and in-hospital mortality, not as causal time-dependent effects or baseline prognostic estimates.

## 3. Results

### 3.1. Study Population and Data Completeness

**Figure 1:**
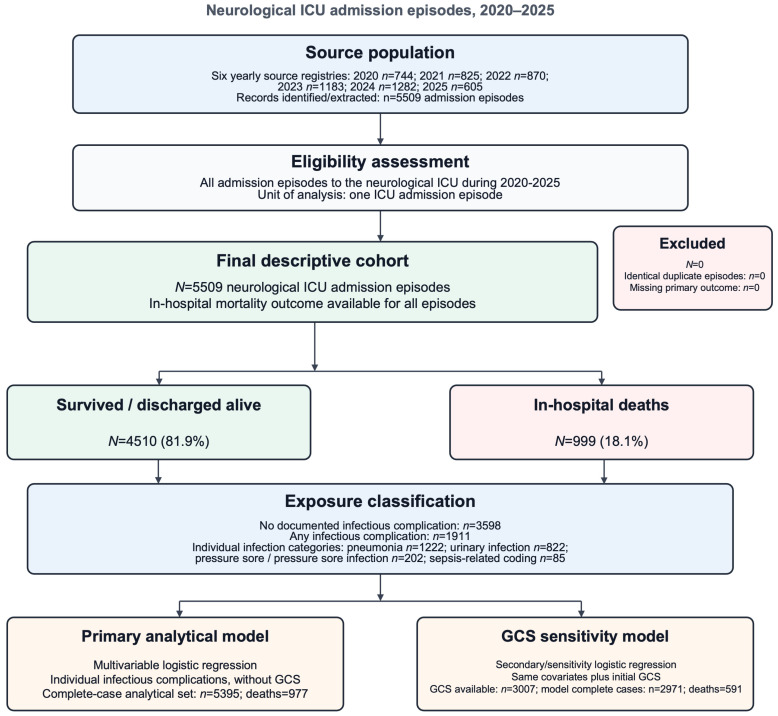
**Cohort flow diagram of neurological ICU admissions included in the retrospective cohort study, 2020–2025.** Note: Counts refer to neurological ICU admissions. Following manual database rechecking, no duplicated patients or recurrent admissions of the same patient were identified; therefore, each analytical record was interpreted as one distinct neurological ICU admission corresponding to one individual patient. The primary model used individual infectious complications without GCS; the GCS model was treated as a sensitivity analysis because GCS was not available for the full cohort.

Across the six annual neurological intensive care unit registers, 5509 ICU admission episodes, rather than unique patients, were included in the final retrospective cohort. In the overall cohort, the median age was 71.0 years (IQR 62.0–80.0). Among episodes with available GCS data, the median initial GCS was 15.0 (IQR 12.0–15.0). The median length of stay was 8.0 days (IQR 5.0–11.0). No admission episodes were excluded after database harmonisation and anonymisation, and in-hospital mortality status was available for all records. Overall, 999 episodes ended in in-hospital death, corresponding to a crude in-hospital mortality of 18.1%; the remaining 4510 episodes were classified as survivor admissions. The cohort selection and analytical flow are shown in Figure 1. All percentages and regression models should therefore be interpreted at the admission-episode level.

Data completeness was high for the primary outcome and most binary clinical, comorbidity, and infection variables. Age was available for 5434 episodes (98.6%), sex for 5484 episodes (99.5%), systolic blood pressure for 4991 episodes (90.6%), and diastolic blood pressure for 4968 episodes (90.2%). Length of stay could be calculated for 5503 episodes (99.9%). By contrast, initial Glasgow Coma Scale (GCS) was available for 3007 episodes (54.6%), supporting its prespecified use in a sensitivity model rather than in the primary model. Variable-level completeness is detailed in Supplementary Table S1.

The multivariable models were therefore fitted as complete-case analyses for the variables included in each model. The primary no-GCS model retained 5395 episodes, whereas the GCS-adjusted sensitivity model retained 2971 episodes, reflecting the lower availability of initial GCS and the additional complete-case requirement for all covariates. A comparison between complete-case episodes and observations excluded from each multivariable model is provided in Supplementary Table S5. In the primary no-GCS model, 5395 admissions were included and 114 were excluded because of incomplete covariate data; mortality was similar between included and excluded observations. In the GCS-adjusted model, 2971 admissions were included and 2538 were excluded, mainly reflecting the lower availability of initial GCS. This larger exclusion reinforces the interpretation of the GCS-adjusted model as a severity-adjusted complete-data sensitivity analysis rather than as the primary full-cohort model.

### 3.2. Baseline and Clinical Characteristics According to In-Hospital Mortality

Admissions ending in in-hospital death involved older patients than survivor admissions, with a median age of 75.0 years (IQR 67.0–83.0) compared with 70.0 years (IQR 61.0–79.0) among survivor admissions (*p* < 0.001). Neurological severity differed substantially in the GCS-available subset: the median initial GCS was 10.0 (IQR 5.0–13.0) among admissions ending in in-hospital death and 15.0 (IQR 14.0–15.0) among survivor admissions (*p* < 0.001). Initial systolic and diastolic blood pressure values did not differ meaningfully between groups. Length of stay showed a statistically significant difference, but this result should be interpreted cautiously because of the skewed distribution and the competing processes of early death and discharge. Length of stay was therefore reported descriptively and was not treated as a primary explanatory variable for mortality (Table 1).

Overall, any infectious complication was documented in 1911 of 5509 ICU admission episodes (34.7%). Pneumonia was the most frequent infectious complication, occurring in 1222 episodes (22.2%), followed by urinary tract infection in 822 episodes (14.9%), pressure sore/pressure sore infection in 202 episodes (3.7%), COVID-related pneumonia in 133 episodes (2.4%), and sepsis-related coding in 85 episodes (1.5%). Infectious complications were markedly more frequent among admissions ending in in-hospital death. Any infectious complication was documented in 711 admissions ending in in-hospital death (71.2%) compared with 1200 survivor admissions (26.6%), corresponding to an unadjusted odds ratio (OR) for in-hospital death of 6.81 (95% CI 5.85–7.93; *p* < 0.001). Pneumonia showed the largest burden among individual infectious complications, being present in 603 admissions ending in in-hospital death (60.4%) and 619 survivor admissions (13.7%) (unadjusted OR 9.60, 95% CI 8.24–11.18; *p* < 0.001). Sepsis-related coding was less frequent in absolute terms but showed the strongest unadjusted association with in-hospital death (6.3% among admissions ending in in-hospital death vs. 0.5% among survivor admissions; unadjusted OR 13.73, 95% CI 8.41–22.42; *p* < 0.001). Urinary tract infection and pressure sore/pressure sore infection were also more frequent among admissions ending in in-hospital death in unadjusted comparisons (Table 2).

Table 2 was retained in the main manuscript because it provides the principal descriptive comparison of neurological diagnoses, comorbidities, and documented infectious complications according to in-hospital mortality status.

The distribution of neurological diagnoses and comorbidities also differed by outcome. Hemorrhagic stroke was more common among admissions ending in in-hospital death than among survivor admissions (30.6% vs. 8.8%; *p* < 0.001), whereas transient ischemic attack and stroke sequelae were more frequent among survivor admissions. Atrial fibrillation, renal disease, hepatic disease, coagulopathy, malignancy, and anticoagulation were all more frequent among admissions ending in in-hospital death in unadjusted analyses. Male sex, hypertension, obesity, and previous stroke were less frequent among admissions ending in in-hospital death than among survivor admissions in unadjusted comparisons, emphasising that crude associations should not be interpreted causally without multivariable adjustment (Table 2).

### 3.3. Annual Distribution of Mortality and Infectious Complications

The annual number of ICU admission episodes ranged from 605 in 2025 to 1282 in 2024. Crude in-hospital mortality was highest in 2021 (190/825, 23.0%) and 2020 (158/744, 21.2%), then decreased to 18.4% in 2022, 16.2% in 2023, 15.6% in 2024, and 16.4% in 2025. The prevalence of any infectious complication increased from 27.0% in 2020 to 32.0% in 2021 and 39.0% in 2022, remaining between 35.3% and 37.4% during 2023–2025. These annual comparisons were descriptive, and no formal temporal trend model was used (Table 3).

Pneumonia accounted for the largest infectious burden in each year, ranging from 17.5% in 2020 to 27.7% in 2022 and 27.6% in 2025. COVID-related pneumonia was recorded only during 2020–2023, with the highest annual proportion in 2021 (5.9%). Urinary tract infection ranged from 11.2% in 2020 to 18.0% in 2024, while sepsis-related coding increased in the later years, reaching 2.8% in 2024 and 2.5% in 2025 (Table 3).

### 3.4. Mortality According to Infectious Complication Status

In unadjusted infection-stratified analyses, mortality was 37.2% among admissions with any infectious complication compared with 8.0% among admissions without documented infection, corresponding to an absolute mortality difference of 29.2 percentage points (unadjusted OR 6.81, 95% CI 5.85–7.93; *p* < 0.001). Pneumonia was associated with a mortality rate of 49.3%, compared with 9.2% in episodes without pneumonia (unadjusted OR 9.60, 95% CI 8.24–11.18; *p* < 0.001). COVID-related pneumonia was associated with a mortality rate of 39.1% (unadjusted OR 3.00, 95% CI 2.10–4.28; *p* < 0.001). Because sepsis-related coding was derived from routine documentation and coding fields, it should be interpreted as documented severe systemic infectious deterioration rather than as formally adjudicated sepsis according to Sepsis-3 criteria. The largest absolute mortality differences were observed for sepsis-related coding and pneumonia (Table 4).

Sepsis-related coding identified the highest-risk infectious subgroup: 63 of 85 episodes with sepsis-related coding ended in death, corresponding to a mortality rate of 74.1%, compared with 17.3% among episodes without sepsis-related coding (unadjusted OR 13.73, 95% CI 8.41–22.42; *p* < 0.001). Pressure sore/pressure sore infection was associated with 40.1% mortality, while urinary tract infection was associated with a smaller but statistically significant crude mortality difference (22.3% vs. 17.4%; *p* = 0.001) (Table 4).

### 3.5. Multivariable Factors Associated with In-Hospital Mortality

**Figure 2:**
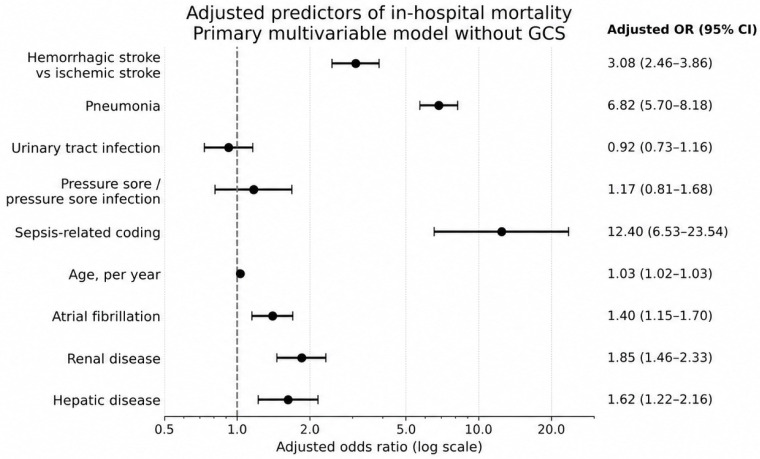
**Forest plot of adjusted factors associated with in-hospital mortality in the primary multivariable model.** Adjusted odds ratios and 95% confidence intervals are shown for the main infectious and clinical factors included in the no-GCS multivariable logistic regression model. The vertical reference line indicates an odds ratio of 1. Values above 1 indicate higher adjusted odds of in-hospital death, whereas values below 1 indicate lower adjusted odds. GCS was not included in this primary model to preserve cohort size and was evaluated separately in a sensitivity model. OR, odds ratio; CI, confidence interval; GCS, Glasgow Coma Scale; UTI, urinary tract infection.

In the primary multivariable logistic regression model, which did not include GCS, pneumonia and sepsis-related coding remained independent factors associated with in-hospital mortality after adjustment for demographic, neurological, comorbidity, and other infectious variables. The adjusted associations from the primary model are visually summarized in Figure 2. Pneumonia was associated with more than sixfold higher adjusted odds of in-hospital death (adjusted OR 6.82, 95% CI 5.70–8.18; *p* < 0.001), while sepsis-related coding, used as a documentation-based marker of severe systemic infectious deterioration, showed the strongest independent association (adjusted OR 12.40, 95% CI 6.53–23.54; *p* < 0.001). In contrast, urinary tract infection (adjusted OR 0.92, 95% CI 0.73–1.16; *p* = 0.487) and pressure sore/pressure sore infection (adjusted OR 1.17, 95% CI 0.81–1.68; *p* = 0.407) were not independently associated with mortality in the primary model (Table 5).

Among non-infectious factors in the primary model, hemorrhagic stroke was independently associated with higher in-hospital mortality compared with ischemic stroke (adjusted OR 3.08, 95% CI 2.46–3.86; *p* < 0.001). Increasing age, atrial fibrillation, renal disease, and hepatic disease also remained independently associated with death. The primary model included 5395 episodes and 977 deaths, with an AUC of 0.885 and a McFadden pseudo-R^2^ of 0.340 (Table 5).

The GCS-adjusted sensitivity model confirmed the robustness of the main infectious associations. After adding initial GCS, pneumonia remained strongly and independently associated with death (adjusted OR 5.25, 95% CI 4.05–6.80; *p* < 0.001), and sepsis-related coding remained strongly associated with mortality (adjusted OR 10.70, 95% CI 4.89–23.40; *p* < 0.001). Initial GCS was itself strongly associated with mortality, with each additional point associated with lower odds of death (adjusted OR 0.78, 95% CI 0.75–0.81; *p* < 0.001). Hemorrhagic stroke, age, atrial fibrillation, diabetes mellitus, and renal disease also remained significantly associated with mortality in this sensitivity model. The GCS-adjusted model included 2971 episodes and 591 deaths and achieved an AUC of 0.920 and a McFadden pseudo-R^2^ of 0.448 (Table 5).

### 3.6. Incremental Mortality-Related Contribution and Model Validation

Adding individual infectious complications to the clinical-only model substantially improved model performance. In the primary cohort without GCS, the AUC increased from 0.826 to 0.885 (ΔAUC +0.058), McFadden pseudo-R^2^ increased from 0.228 to 0.340 (ΔR^2^ +0.112), and AIC decreased from 3984.8 to 3421.2 (ΔAIC −563.6). The likelihood-ratio test strongly supported the incremental contribution of infectious complications (LR χ^2^ = 571.6, df = 4; *p* < 0.001). In the GCS-available cohort, adding infectious complications increased the AUC from 0.890 to 0.920 (ΔAUC +0.030), improved McFadden pseudo-R^2^ from 0.377 to 0.448, and reduced AIC by 204.8 points (LR χ^2^ = 212.8, df = 4; *p* < 0.001) (Table 6).

Internal validation and calibration analyses supported the stability of the modelling results. The primary model showed an apparent AUC of 0.8846 and a bootstrap-corrected AUC of 0.8795, with a Brier score of 0.0984 and a bootstrap-corrected calibration slope of 0.9670. The GCS-adjusted sensitivity model showed higher discrimination, with an apparent AUC of 0.9198 and a bootstrap-corrected AUC of 0.9147; the Brier score was 0.0841 and the bootstrap-corrected calibration slope was 0.9505. The Hosmer-Lemeshow test was significant in the primary model but not in the GCS-adjusted sensitivity model; given the large sample size, calibration was interpreted in conjunction with the Brier score, calibration slope, and bootstrap-corrected estimates (Supplementary Table S2).

No relevant multicollinearity was detected in either multivariable model. The maximum variance inflation factor was 2.157 in the primary no-GCS model and 2.364 in the GCS-adjusted sensitivity model, with minimum tolerance values of 0.464 and 0.423, respectively (Supplementary Table S6).

Calibration curves for the primary no-GCS model and the GCS-adjusted sensitivity model are provided in Supplementary Figures S1 and S2. Decision curve analysis showed that the clinical-plus-infections models provided higher net benefit than the corresponding clinical-only models across clinically relevant threshold-probability ranges in both the primary no-GCS cohort and the GCS-available cohort (Supplementary Figures S3 and S4).

### 3.7. COVID-Related Sensitivity Analysis

Because COVID-related pneumonia represented a specific pandemic-era infectious phenotype, a dedicated sensitivity analysis was performed. Mortality was 9.2% among episodes without pneumonia, 50.6% among episodes with non-COVID pneumonia, and 39.1% among episodes with COVID-related pneumonia (Supplementary Table S3).

The association between pneumonia and in-hospital mortality was not explained exclusively by COVID-related pneumonia. After excluding COVID-related pneumonia cases, pneumonia remained independently associated with mortality in the no-GCS model (adjusted OR 7.43, 95% CI 6.15–8.98; *p* < 0.001) and in the GCS-adjusted model (adjusted OR 5.66, 95% CI 4.31–7.42; *p* < 0.001). In models separating pneumonia categories, non-COVID pneumonia remained strong independent associates with mortality both without GCS (adjusted OR 7.49, 95% CI 6.20–9.04; *p* < 0.001) and with GCS adjustment (adjusted OR 5.67, 95% CI 4.33–7.44; *p* < 0.001). COVID-related pneumonia was also independently associated with mortality, although with lower effect estimates than non-COVID pneumonia, both in the no-GCS model (adjusted OR 3.43, 95% CI 2.25–5.24; *p* < 0.001) and the GCS-adjusted model (adjusted OR 2.57, 95% CI 1.28–5.16; *p* = 0.0077). Across COVID sensitivity models, sepsis-related coding remained independently associated with death, whereas urinary tract infection and pressure sore/pressure sore infection did not show independent mortality associations (Supplementary Table S4).

## 4. Discussion

### 4.1. Principal Findings

In this six-year retrospective neurological ICU cohort, documented infectious complications were frequent and were strongly associated with in-hospital mortality. Pneumonia represented the dominant mortality-associated infectious complication, while sepsis-related coding identified a smaller but particularly high-risk subgroup. By contrast, urinary tract infection and pressure sore or pressure sore-related infection showed crude mortality associations but did not retain independent associations after multivariable adjustment [1,3,5,6,15].

These findings support three main interpretations. First, infectious complications are not merely descriptive hospital-course events in neurological intensive care, but are associated with clinically meaningful mortality differences. Second, not all infection phenotypes showed the same strength of mortality association: pneumonia and sepsis-related coding appear to identify a more severe mortality-risk signal than urinary tract infection or pressure sore-related infection. Third, the association between infection and mortality persisted after adjustment for neurological severity in the GCS-available subset, suggesting that the infection–mortality relationship was not explained solely by baseline neurological impairment [1,3,5,6,15].

Importantly, these findings should be interpreted in the context of the hospital-course nature of the infectious variables. Pneumonia, urinary tract infection, pressure sore or pressure sore-related infection, and sepsis-related coding were documented during hospitalization and were not baseline exposures measured at ICU admission. Therefore, the observed estimates indicate associations with in-hospital mortality rather than causal effects or classical baseline prognostic effects.

The incremental model analysis further strengthens this interpretation. Adding individual infectious complications to clinical-only models improved discrimination and model fit both in the primary cohort and in the GCS-available subset. This suggests that infectious complications provide additional mortality-related information beyond demographic characteristics, neurological diagnosis, comorbidity burden, and, where available, initial GCS. From a clinical standpoint, these findings reinforce the importance of systematic infection surveillance and early recognition of infectious deterioration in neurological intensive care practice [16,17,18].

### 4.2. Pneumonia as the Dominant Infectious Complication Associated with Mortality

Pneumonia was the most important infectious complication in the present study, both in terms of frequency and adjusted association with in-hospital mortality. This finding is consistent with previous literature showing that pneumonia is one of the leading healthcare-associated infections in neurological ICUs and one of the most clinically consequential infectious complications after acute stroke [1,3,6,8,10,19]. In the present cohort, pneumonia was associated with markedly higher crude mortality and remained independently associated with mortality after adjustment for demographic factors, neurological diagnosis, comorbidities, and other infection variables.

The strong mortality signal associated with pneumonia is biologically and clinically plausible in neurocritical care. Patients with severe neurological injury frequently have impaired swallowing, reduced cough reflex, altered consciousness, aspiration risk, mechanical ventilation exposure, immobility, and impaired airway clearance. These factors create a particularly favorable context for lower respiratory tract infection. At the same time, pneumonia may amplify neurological and systemic deterioration through hypoxemia, systemic inflammation, fever, hemodynamic instability, increased metabolic demand, and delayed rehabilitation. Therefore, pneumonia may function both as a consequence of neurological severity and as an active contributor to clinical deterioration [8,9,10,19,20,21].

At the same time, the present analysis cannot determine whether pneumonia directly contributed to death, reflected more severe neurological deterioration, or both. Because infection onset dates and detailed ICU intervention variables were not uniformly available, pneumonia may partly represent a marker of longer ICU exposure, impaired consciousness, aspiration risk, mechanical ventilation exposure, or progressive neurological decline. Therefore, the strong association between pneumonia and mortality should be interpreted as a clinically important mortality-related signal rather than as proof of an independent causal effect.

The present findings also align with the stroke-associated pneumonia literature. The Pneumonia in Stroke Consensus Group emphasized that lower respiratory tract infections after stroke require standardized terminology and diagnostic criteria because clinical and radiological interpretation may be difficult in neurologically impaired patients [8]. The PISCES recommendations further highlighted the complexity of antibiotic treatment decisions in stroke-associated pneumonia, where the risks of delayed treatment must be balanced against diagnostic uncertainty and antimicrobial stewardship [9]. More recent meta-analytic evidence confirms that post-stroke pneumonia is associated not only with increased in-hospital mortality but also with longer-term mortality and worse functional outcomes (10). Although the present study was not limited to stroke-associated pneumonia and included a broader neurological ICU cohort, the persistent association between pneumonia and mortality is consistent with this wider evidence base [6,8,9,10,19,20,21].

An important aspect of the present study is that the pneumonia–mortality association was not explained exclusively by COVID-related pneumonia. Non-COVID pneumonia remained strongly associated with mortality after exclusion of COVID-related pneumonia cases and in models separating non-COVID pneumonia from COVID-related pneumonia. This is clinically relevant because it indicates that the observed pneumonia signal reflects a broader neurocritical care phenomenon rather than only a pandemic-era effect. Pneumonia should therefore remain a central target for surveillance, prevention, early diagnosis, and treatment in neurological ICU populations beyond the specific context of COVID-19 [1,8,9,10,19,20,21].

### 4.3. Sepsis-Related Coding and Systemic Infectious Deterioration

Sepsis-related coding showed the strongest adjusted association with in-hospital mortality in the present cohort. This finding should not be interpreted as evidence that formally adjudicated sepsis per se was measured in this cohort; rather, the variable captures routine clinical documentation suggestive of severe infection-related systemic deterioration. Although sepsis-related documentation was less frequent than pneumonia or urinary tract infection, it identified a subgroup with very high crude mortality and remained independently associated with death in the primary, GCS-adjusted, and COVID-related sensitivity models. This pattern suggests that sepsis-related coding captured a severe systemic infectious phenotype rather than a simple infection label [22,23].

This finding should be interpreted carefully. In this retrospective database, sepsis was not formally adjudicated using standardized criteria, organ dysfunction scoring, microbiological confirmation, source-control data, or precise timing of infectious onset. Therefore, the variable was deliberately analyzed and described as “sepsis-related coding” rather than as formally adjudicated sepsis. This distinction is important because contemporary sepsis definitions emphasize life-threatening organ dysfunction caused by a dysregulated host response to infection, which requires clinical and physiological information that may not be uniformly available in routine retrospective datasets [22].

Nevertheless, the strong association between sepsis-related coding and mortality is clinically meaningful. In neurological ICU patients, systemic infectious deterioration may be especially difficult to separate from neurological decline, sedation, impaired consciousness, dysautonomia, or respiratory failure. When sepsis-related wording appears in routine documentation, it may therefore represent advanced systemic deterioration, high clinician concern, or a severe infection phenotype. The high adjusted odds ratios observed in the present study support the use of sepsis-related coding as a high-risk marker, while also emphasizing the need for caution in causal interpretation [22,23].

Future studies should attempt to validate sepsis-related events prospectively using standardized sepsis definitions, infection source adjudication, organ dysfunction scores, microbiological data, and timing of onset. Such work would help distinguish whether the excess mortality associated with sepsis-related coding reflects infection severity itself, delayed recognition, baseline neurological severity, systemic organ failure, or a combination of these mechanisms [22,23].

### 4.4. Urinary Tract Infection and Pressure Sore-Related Infection: Unadjusted Versus Adjusted Associations

Urinary tract infection and pressure sore or pressure sore-related infection were more frequent among admissions ending in in-hospital death in unadjusted analyses, but neither remained independently associated with in-hospital mortality after multivariable adjustment. This distinction is important. The crude associations suggest that these complications occur more often during more severe hospital-course trajectories, but the adjusted models suggest that their apparent mortality relationship may be largely explained by age, neurological diagnosis, comorbidity burden, concurrent infections, and overall clinical severity [11,12,24].

Urinary tract infection is common in neurological patients because of bladder dysfunction, reduced mobility, older age, comorbid disease, and urinary catheter exposure [11]. In clinical practice, urinary tract infection may contribute to fever, delirium, systemic inflammatory response, prolonged hospitalization, and increased care complexity. However, compared with pneumonia and sepsis-related coding, urinary tract infection may less often represent a direct driver of early in-hospital death, particularly after adjustment for other clinical and infectious covariates. The present findings are consistent with this interpretation: urinary tract infection showed a statistically significant unadjusted association with death, but not an independent adjusted association [11].

A similar interpretation applies to pressure sore and pressure sore-related infection. Pressure injuries are clinically important complications of immobility, critical illness, impaired perfusion, nutritional vulnerability, and prolonged care dependency. Large ICU-level data have shown that pressure injuries are common in adult ICU patients and that increasing pressure injury severity is associated with mortality [12]. In the present study, pressure sore or pressure sore-related infection was associated with substantially higher crude mortality, but this association was attenuated after adjustment. This suggests that pressure sore-related documentation may operate as a marker of frailty, immobility, prolonged severity, and complex hospital course rather than as an independent factor associated with mortality in the available model [12,24].

These findings should not be interpreted as minimizing the clinical importance of urinary tract infection or pressure sore-related infection. Both complications remain relevant for patient comfort, quality of care, antimicrobial use, nursing workload, rehabilitation delay, and institutional infection-prevention programs. Rather, the results indicate that, in this mortality-focused analysis, pneumonia and sepsis-related coding carried a more robust independent association with death than urinary tract infection or pressure sore-related infection [11,12,24].

### 4.5. Neurological Severity, GCS, and Robustness of the Infection–Mortality Signal

Neurological severity is a major determinant of outcome in neurocritical care and represents an essential potential confounder in analyses of infectious complications. Patients with lower consciousness levels are more likely to aspirate, require airway support, remain immobilized, receive invasive devices, and develop infectious complications. At the same time, they are also more likely to die because of the severity of the primary neurological insult. For this reason, evaluating whether infection-related associations persist after adjustment for neurological severity is methodologically important [1,8,25].

In the present study, initial GCS was available in approximately half of the cohort and was therefore not included in the primary model. This decision preserved almost the full cohort for the main analysis and avoided restricting the primary conclusions to a subset with available neurological severity data. However, a dedicated GCS-adjusted sensitivity model was performed. As expected, initial GCS was strongly and independently associated with mortality, with higher GCS values corresponding to lower odds of death. This confirms the clinical validity of GCS as a marker of neurological severity in this cohort [25].

Importantly, pneumonia and sepsis-related coding remained independently associated with in-hospital mortality after adding GCS to the model. The effect estimates were attenuated compared with the primary model, which is expected because part of the infection risk is linked to neurological severity. However, the associations remained strong and statistically significant. This supports the robustness of the main finding: the mortality association of pneumonia and sepsis-related coding was not explained solely by impaired consciousness or baseline neurological severity [1,8,22,25].

The GCS-adjusted model also showed improved discrimination compared with the primary model, which is consistent with the importance of neurological severity in mortality-risk assessment. However, because GCS was missing in a substantial proportion of records, this model should be interpreted as a sensitivity analysis rather than as the primary analytical framework. The concordance between the primary and GCS-adjusted models strengthens the internal consistency of the study findings [16,17,18,25].

### 4.6. Incremental Mortality-Related Value of Infectious Complications

Beyond the adjusted associations of individual infection variables, the incremental model analysis showed that infectious complications improved mortality risk stratification when added to clinical-only models. In the primary cohort, the inclusion of individual infectious complications increased the area under the receiver operating characteristic curve, improved McFadden pseudo-R^2^, and substantially reduced the Akaike information criterion. A similar pattern was observed in the GCS-available subset, despite the already strong contribution of neurological severity to mortality-risk assessment. These findings suggest that infectious complications provide mortality-related information that is not fully captured by demographic characteristics, neurological diagnosis, comorbidity burden, calendar year, or GCS [16,17,18,26,27]. The decision curve analysis further supported the incremental value of adding documented infectious complications to clinical-only models, showing higher net benefit for the clinical-plus-infections models across clinically relevant threshold-probability ranges. However, this analysis should be interpreted as exploratory. Because infectious complications were hospital-course variables and infection timing was unavailable, the decision curve analysis does not establish that infection variables could be used as admission-time decision tools. Rather, it supports their potential value for dynamic in-hospital risk assessment and clinical awareness during the ICU course.

This result is clinically relevant because neurological ICU mortality is often conceptualized primarily through the severity of the initial neurological insult. While neurological severity is clearly central, the present findings indicate that hospital-course infectious complications add measurable mortality-related information. Pneumonia and sepsis-related coding were particularly informative in this regard. Their inclusion improved model performance even after accounting for major clinical variables, supporting the view that infection surveillance is not only a quality-of-care issue but also an important component of mortality-risk assessment in neurological intensive care [1,3,5,15,16].

The internal validation and calibration findings support the stability of the models. The primary model showed high discrimination, and the bootstrap-corrected estimates suggested limited optimism. The GCS-adjusted sensitivity model showed even higher discrimination and acceptable calibration, consistent with the major role of neurological severity in mortality risk. The significant Hosmer–Lemeshow test in the primary model should be interpreted cautiously because this test is highly sensitive to large sample size; therefore, calibration was more appropriately assessed together with the Brier score, calibration slope, and bootstrap-corrected estimates. Taken together, these analyses suggest that the observed infection–mortality signal was not an artefact of a single model specification [16,17,18,26,27].

### 4.7. COVID-Related Sensitivity Analysis

The study period included the COVID-19 pandemic, making it necessary to evaluate whether the association between pneumonia and in-hospital mortality was driven primarily by COVID-related pneumonia. This was particularly important because COVID-related pneumonia represents a distinct infectious phenotype with specific pathophysiological, epidemiological, and healthcare-system implications. If the overall pneumonia signal had been driven mainly by COVID-related cases, the interpretation of the study would have been more limited to the pandemic period [28,29].

The COVID-related sensitivity analyses showed that this was not the case. Non-COVID pneumonia remained strongly associated with in-hospital mortality after exclusion of COVID-related pneumonia cases and in models separating pneumonia categories. COVID-related pneumonia was also independently associated with mortality, but its effect estimates were lower than those observed for non-COVID pneumonia. This finding supports the robustness of pneumonia as a mortality-associated clinical marker in neurological ICU patients and indicates that the pneumonia–mortality relationship reflects a broader neurocritical care phenomenon rather than a pandemic-specific artefact [1,6,8,9,19,20,21,28,29].

The higher crude mortality observed among admissions with non-COVID pneumonia compared with COVID-related pneumonia should not be interpreted as evidence that non-COVID pneumonia is intrinsically more lethal than COVID-related pneumonia. In this neurological ICU cohort, non-COVID pneumonia may have included aspiration pneumonia, hospital-acquired pneumonia, ventilator-associated pneumonia when ventilatory support was used, or pneumonia occurring in patients with severe neurological impairment and prolonged immobilization. Because detailed information on infection onset, intubation, duration of mechanical ventilation, respiratory support, and other ICU interventions was not uniformly available, this comparison should be interpreted as a descriptive mortality pattern rather than a causal comparison between pneumonia phenotypes.

These results are consistent with the general neurocritical care and stroke literature, in which pneumonia has long been recognized as a frequent and clinically consequential complication outside the COVID-19 context [1,6,8,10,19,20,21]. The persistence of the pneumonia signal after COVID-related sensitivity analyses strengthens the external relevance of the study findings. It also supports the continued prioritization of pneumonia prevention, aspiration-risk assessment, respiratory monitoring, early diagnosis, and appropriate antimicrobial stewardship in neurological ICU practice beyond pandemic-specific care pathways [2,9,23,24,28,29].

### 4.8. Romanian and Institutional Relevance

The present study has particular relevance in the Romanian healthcare context, where healthcare-associated infection surveillance and reporting have undergone substantial legislative and institutional evolution. Romanian analyses have emphasized that, despite regulatory progress, under-reporting, infrastructure constraints, and implementation barriers continue to affect the surveillance and interpretation of healthcare-associated infections [13]. In this context, detailed institutional analyses may provide useful complementary evidence by describing local infection patterns, documentation practices, and outcome associations in specific high-risk clinical settings [13,14,30].

Neurological intensive care represents one such high-risk setting. Patients admitted to neurological ICUs may have prolonged immobilization, impaired airway protection, swallowing dysfunction, altered consciousness, and frequent exposure to invasive devices. These vulnerabilities make infection prevention and recognition particularly important. However, Romanian data focusing specifically on neurological ICU infection burden and mortality-risk assessment remain limited. Most available evidence is either general ICU surveillance, general healthcare-associated infection reporting, or stroke-focused literature rather than full-cohort neurological ICU outcome modeling [13,14,30].

This study also extends previous institutional work. A recent Romanian neurological ICU study described healthcare-associated infections among deceased stroke patients and found pneumonia to be the dominant infectious complication in that fatal subgroup [14]. However, because that analysis was restricted to deceased patients, it could not compare infection patterns across mortality-outcome groups or determine whether individual infectious complications were independently associated with mortality. The present study addresses this limitation by analyzing all available neurological ICU admission episodes recorded over a six-year period, including both survivor admissions and admissions ending in in-hospital death. This mortality-outcome comparison framework is essential for estimating mortality associations and for evaluating the incremental mortality-related value of infections [14].

From an institutional perspective, the findings may support targeted quality-improvement efforts. The strong and robust association between pneumonia and mortality suggests that pneumonia surveillance, dysphagia and aspiration-risk assessment, respiratory care protocols, mobilization when feasible, and early recognition of respiratory deterioration should remain central priorities in neurological ICU care. The high-risk profile associated with sepsis-related coding also indicates the need for timely recognition of systemic infectious deterioration and careful documentation of infection source, organ dysfunction, microbiology, and treatment timing [2,8,9,22,23].

The external validity of these findings should be considered in relation to local ICU organization, infection surveillance practices, documentation standards, antimicrobial stewardship implementation, staffing patterns, respiratory support pathways, and access to microbiological testing. Therefore, the results may be most directly applicable to neurological ICU settings with similar documentation systems, case-mix, and institutional organization. At the same time, the study provides a useful framework for Romanian and regional neurological ICU services seeking to evaluate infection-related mortality associations, improve documentation quality, and develop surveillance-compatible outcome analyses.

### 4.9. Strengths and Limitations

This study has several strengths. First, it included a large six-year cohort of neurological ICU admission episodes, allowing analysis across a substantial institutional experience rather than a narrow diagnostic subgroup. Second, the study used a full-cohort survivor-versus-non-survivor design, which allowed direct evaluation of infectious complications as factors associated with in-hospital mortality. Third, individual infectious complications were analyzed separately, rather than being collapsed only into a composite infection variable. This allowed the study to distinguish the strong adjusted mortality associations of pneumonia and sepsis-related coding from the more attenuated adjusted associations of urinary tract infection and pressure sore-related infection.

Fourth, the analysis included a prespecified primary model without GCS and a GCS-adjusted sensitivity model. This approach balanced cohort preservation with the need to account for neurological severity. Fifth, the study evaluated the incremental mortality-related value of infections beyond baseline clinical variables and performed internal validation and calibration analyses. Finally, the COVID-related sensitivity analysis strengthened the interpretation of the pneumonia signal by showing that it was not driven exclusively by COVID-related pneumonia [16,17,18,26,27].

Several limitations must also be acknowledged. The study was retrospective and single-center, which limits causal inference and may affect generalizability. Infection variables were derived from routinely collected clinical documentation and coding fields rather than from prospective standardized infection adjudication. As a result, misclassification, under-documentation, and variation in diagnostic thresholds are possible. Some comorbidity variables, including documented obesity, may have been influenced by routine documentation practices and should therefore be interpreted as recorded clinical documentation variables rather than systematically adjudicated comorbidity endpoints. Sepsis was analyzed as sepsis-related coding rather than formally adjudicated sepsis, because the dataset did not consistently contain the standardized information needed to apply formal sepsis definitions retrospectively [22,23]. Therefore, this variable may combine true sepsis, suspected sepsis, clinician-perceived systemic infectious deterioration, and documentation practices, and should be interpreted as a high-risk clinical documentation marker rather than a pathophysiologically confirmed sepsis endpoint.

Residual confounding by ICU interventions is also important. Detailed data on intubation, duration of mechanical ventilation, tracheostomy, central venous catheter exposure, urinary catheter duration, vasopressor use, enteral or parenteral feeding, and other ICU support measures were not uniformly available. These variables may influence both the occurrence of infectious complications and the risk of death. Consequently, the adjusted models could not fully account for intervention-related exposure, device-related infection risk, or overall physiological severity during the ICU stay.

The dataset was structured at the neurological ICU admission level. Following reviewer comments, the database was manually rechecked for duplicate or recurrent patient entries, and no duplicated patients or recurrent admissions of the same patient were identified. Therefore, each analytical record was interpreted as one distinct neurological ICU admission corresponding to one individual patient. GCS was available in only approximately half of the cohort. For this reason, it was not included in the primary model, and the GCS-adjusted model was interpreted as a severity-adjusted complete-data sensitivity analysis. Although the consistency of the findings across both models supports robustness, residual confounding by neurological severity remains possible [25]. In addition, global physiological severity scores such as APACHE II, SOFA, and SAPS II were not uniformly available, limiting adjustment for overall critical illness severity beyond neurological diagnosis, comorbidities, age, and the GCS-available sensitivity analysis.

A major limitation is the absence of consistently available timing for infection onset. Infectious complications were hospital-course variables, and their exact onset relative to ICU admission, neurological deterioration, exposure to invasive devices, respiratory support, or death could not be reliably reconstructed. This creates a risk of time-dependent bias because admissions with longer survival or longer hospitalization had more opportunity for pneumonia, urinary tract infection, pressure sore-related complications, or sepsis-related coding to be documented. Therefore, the observed associations may partly reflect longer exposure time, greater neurological severity, prolonged ICU dependency, or terminal deterioration rather than a direct biological effect of infection itself.

For this reason, Cox regression or Kaplan–Meier analysis was not used as a primary analytical framework, because treating infectious complications as fixed baseline exposures would risk immortal time bias. The logistic regression approach was appropriate for estimating associations with in-hospital mortality in the available dataset, but it does not establish temporal sequence or causality. Future prospective studies with infection-onset dates and time-dependent exposure modeling are needed to clarify whether infectious complications contribute causally to mortality, act primarily as markers of clinical deterioration, or both.

### 4.10. Future Directions

Future research should prospectively evaluate infectious complications in neurological ICU populations using standardized definitions, systematic infection surveillance, and consistent recording of infection-onset dates. Prospective designs should also collect device exposure, respiratory support variables, antimicrobial timing, microbiological confirmation, source-control data, dysphagia screening results, and standardized neurological and physiological severity scores. Such data would allow time-dependent analyses and better separation of causal effects, severity markers, and terminal hospital-course events [8,9,21,31].

Multicenter studies would also be valuable to determine whether the observed infection–mortality associations are reproducible across different neurological ICU settings and healthcare systems, and to support benchmarking of infection-related outcomes in neurocritical care [8,9,22,23,25].

At the institutional level, the present findings support the development of targeted surveillance and prevention strategies focused particularly on pneumonia and systemic infectious deterioration. Future quality-improvement projects could evaluate aspiration-prevention bundles, early dysphagia assessment, respiratory care protocols, device-use optimization, mobilization strategies, antimicrobial stewardship, and standardized documentation of infection-related organ dysfunction. Multicenter Romanian studies would also be valuable to determine whether the observed findings are reproducible across different neurological ICU settings and to support national benchmarking of infection-related outcomes in neurocritical care [2,23,24,28,29,30].

Ultimately, infection surveillance in neurological intensive care should not be viewed only as an administrative or epidemiological requirement. The present findings suggest that infectious complications, especially pneumonia and sepsis-related coding, may provide clinically meaningful mortality-related information and should be integrated into outcome assessment, risk stratification, and quality-improvement frameworks for neurological ICU patients [1,2,3,5,15,16,17,18].

## 5. Conclusions

In this six-year retrospective cohort of neurological intensive care admissions, documented hospital-course infectious complications were strongly associated with in-hospital mortality. Pneumonia represented the dominant infectious complication and remained independently associated with death after adjustment for demographic characteristics, neurological diagnosis, comorbidities, and, in sensitivity analysis, initial Glasgow Coma Scale score. Sepsis-related coding, interpreted as a documentation-based marker of severe systemic infectious deterioration rather than formally adjudicated sepsis, identified a smaller but particularly high-risk subgroup and showed the strongest adjusted association with in-hospital mortality.

The incremental model analysis demonstrated that infectious complications added meaningful mortality-related information beyond baseline clinical and neurological variables. This association was not explained exclusively by COVID-related pneumonia, as non-COVID pneumonia remained robustly associated with mortality in dedicated sensitivity analyses.

These findings support the clinical relevance of systematic infection surveillance, early recognition of pneumonia and sepsis-related deterioration, and integration of hospital-course infectious complications into mortality-risk assessment in neurological intensive care. Given the retrospective, single-center design and the absence of standardized infection-onset timing, the results should be interpreted as evidence of strong mortality association rather than proof of causality. Future prospective studies should evaluate standardized infection definitions, temporal relationships between infection onset and clinical deterioration, and the impact of targeted prevention and early-treatment strategies on outcomes in neurological intensive care populations.

## Figures and Tables

**Table 1:** Continuous baseline and clinical variables according to in-hospital mortality status.

Variable	N Available	Survivors	Deaths	*p* Value	Test
Age, years	5434	N = 4447; median (IQR) 70.0 (61.0–79.0); mean ± SD 68.8 ± 13.2	N = 987; median (IQR) 75.0 (67.0–83.0); mean ± SD 73.9 ± 12.3	<0.001	Mann-Whitney U
Initial GCS	3007	N = 2408; median (IQR) 15.0 (14.0–15.0); mean ± SD 14.0 ± 3.2	N = 599; median (IQR) 10.0 (5.0–13.0); mean ± SD 9.4 ± 4.3	<0.001	Mann-Whitney U
Initial systolic BP, mmHg	4991	N = 4112; median (IQR) 154.0 (136.0–174.0); mean ± SD 155.4 ± 33.9	N = 879; median (IQR) 156.0 (130.0–180.0); mean ± SD 156.1 ± 40.7	0.898	Mann-Whitney U
Initial diastolic BP, mmHg	4968	N = 4094; median (IQR) 84.0 (75.0–96.0); mean ± SD 85.1 ± 19.1	N = 874; median (IQR) 84.0 (72.0–100.0); mean ± SD 85.5 ± 21.6	0.829	Mann-Whitney U
Length of stay, days	5503	N = 4505; median (IQR) 8.0 (5.0–11.0); mean ± SD 9.2 ± 36.4	N = 998; median (IQR) 7.0 (4.0–11.0); mean ± SD 7.5 ± 29.2	<0.001	Mann-Whitney U

Note: Continuous variables are reported as median (interquartile range) and mean ± standard deviation. *p* values were calculated using the Mann-Whitney U test. Length of stay should be interpreted cautiously because of skewed distribution and the competing processes of early death and discharge.

**Table 2:** Categorical baseline, neurological, comorbidity, and infectious variables according to in-hospital mortality status.

Variable	N Available	Survivor Admissions n (%)	In-Hospital Death Admissions n (%)	Unadjusted OR (95% CI)	*p* Value
Male sex	5484	2452 (54.6%)	489 (49.2%)	0.80 (0.70–0.92)	0.002
Any infectious complication	5509	1200 (26.6%)	711 (71.2%)	6.81 (5.85–7.93)	<0.001
Pneumonia	5508	619 (13.7%)	603 (60.4%)	9.60 (8.24–11.18)	<0.001
COVID-related pneumonia	5509	81 (1.8%)	52 (5.2%)	3.00 (2.10–4.28)	<0.001
Urinary tract infection	5508	639 (14.2%)	183 (18.3%)	1.36 (1.13–1.63)	0.001
Pressure sore/pressure sore infection	5505	121 (2.7%)	81 (8.1%)	3.21 (2.40–4.30)	<0.001
Sepsis-related coding	5509	22 (0.5%)	63 (6.3%)	13.73 (8.41–22.42)	<0.001
Ischemic stroke documented	5509	3050 (67.6%)	673 (67.4%)	0.99 (0.85–1.14)	0.903
Hemorrhagic stroke documented	5509	399 (8.8%)	306 (30.6%)	4.55 (3.84–5.39)	<0.001
TIA documented	5509	553 (12.3%)	7 (0.7%)	0.05 (0.02–0.11)	<0.001
Stroke sequelae documented	5508	654 (14.5%)	47 (4.7%)	0.29 (0.21–0.39)	<0.001
Hypertension	5508	3842 (85.2%)	805 (80.6%)	0.72 (0.60–0.86)	<0.001
Ischemic heart disease/prior MI	5508	2423 (53.7%)	572 (57.3%)	1.15 (1.00–1.32)	0.047
Atrial fibrillation	5508	1060 (23.5%)	379 (37.9%)	1.99 (1.72–2.30)	<0.001
Diabetes mellitus	5508	1026 (22.8%)	238 (23.8%)	1.06 (0.90–1.25)	0.493
Obesity	5506	2967 (65.8%)	246 (24.6%)	0.17 (0.14–0.20)	<0.001
Renal disease	5509	422 (9.4%)	221 (22.1%)	2.75 (2.30–3.29)	<0.001
Hepatic disease	5508	346 (7.7%)	138 (13.8%)	1.93 (1.56–2.38)	<0.001
Coagulopathy	5509	182 (4.0%)	91 (9.1%)	2.38 (1.83–3.10)	<0.001
Previous stroke	5509	1046 (23.2%)	164 (16.4%)	0.65 (0.54–0.78)	<0.001
Malignancy	5509	348 (7.7%)	100 (10.0%)	1.33 (1.05–1.68)	0.019
Anticoagulation	5509	30 (0.7%)	16 (1.6%)	2.43 (1.32–4.48)	0.006

Note: Categorical variables are reported as n (%). Percentages are calculated within the survivor and death groups, respectively. OR values are unadjusted odds ratios for in-hospital death and should not be interpreted as causal estimates. Adjusted interpretation should rely on the multivariable models.

**Table 3:** Annual distribution of admission episodes, in-hospital mortality, and infectious complications.

Year	Episodes	Deaths n (%)	Any Infection n (%)	Pneumonia n (%)	COVID Pneumonia n (%)	UTI n (%)	Pressure Sore/Infection n (%)	Sepsis-Related n (%)
2020	744	158 (21.2%)	201 (27.0%)	130 (17.5%)	26 (3.5%)	83 (11.2%)	26 (3.5%)	1 (0.1%)
2021	825	190 (23.0%)	264 (32.0%)	164 (19.9%)	49 (5.9%)	104 (12.6%)	17 (2.1%)	16 (1.9%)
2022	870	160 (18.4%)	339 (39.0%)	241 (27.7%)	40 (4.6%)	129 (14.8%)	30 (3.4%)	0 (0.0%)
2023	1183	192 (16.2%)	428 (36.2%)	259 (21.9%)	18 (1.5%)	188 (15.9%)	47 (4.0%)	17 (1.4%)
2024	1282	200 (15.6%)	453 (35.3%)	261 (20.4%)	0 (0.0%)	231 (18.0%)	60 (4.7%)	36 (2.8%)
2025	605	99 (16.4%)	226 (37.4%)	167 (27.6%)	0 (0.0%)	87 (14.4%)	22 (3.6%)	15 (2.5%)

Note: Percentages are calculated within each year.

**Table 4:** In-hospital mortality according to infectious complication status.

Infection Variable	Exposed N	Deaths Among Exposed	Mortality Exposed	Unexposed N	Deaths Among Unexposed	Mortality Unexposed	Absolute Mortality Difference	Unadjusted OR (95% CI), *p* Value
Any infectious complication	1911	711	37.2%	3598	288	8.0%	+29.2 pp	6.81 (5.85–7.93), *p* < 0.001
Pneumonia	1222	603	49.3%	4286	395	9.2%	+40.1 pp	9.60 (8.24–11.18), *p* < 0.001
COVID-related pneumonia	133	52	39.1%	5376	947	17.6%	+21.5 pp	3.00 (2.10–4.28), *p* < 0.001
Urinary tract infection	822	183	22.3%	4686	816	17.4%	+4.9 pp	1.36 (1.13–1.63), *p* = 0.001
Pressure sore/pressure sore infection	202	81	40.1%	5303	914	17.2%	+22.9 pp	3.21 (2.40–4.30), *p* < 0.001
Sepsis-related coding	85	63	74.1%	5424	936	17.3%	+56.8 pp	13.73 (8.41–22.42), *p* < 0.001

Note: Mortality comparisons are unadjusted. Absolute mortality difference was calculated as mortality among exposed admissions minus mortality among unexposed admissions and is reported in percentage points (pp). Adjusted interpretation should rely on the multivariable logistic regression models.

**Table 5:** Multivariable logistic regression for in-hospital mortality: primary and GCS-adjusted sensitivity models.

Model	Variable	Adjusted OR (95% CI)	*p* Value
Primary model, no GCS	Hemorrhagic stroke vs. ischemic stroke	3.08 (2.46–3.86)	<0.001
Primary model, no GCS	Pneumonia	6.82 (5.70–8.18)	<0.001
Primary model, no GCS	Urinary tract infection	0.92 (0.73–1.16)	0.487
Primary model, no GCS	Pressure sore/pressure sore infection	1.17 (0.81–1.68)	0.407
Primary model, no GCS	Sepsis-related coding	12.40 (6.53–23.54)	<0.001
Primary model, no GCS	Age, per year	1.03 (1.02–1.03)	<0.001
Primary model, no GCS	Atrial fibrillation	1.40 (1.15–1.70)	<0.001
Primary model, no GCS	Renal disease	1.85 (1.46–2.33)	<0.001
Primary model, no GCS	Hepatic disease	1.62 (1.22–2.16)	<0.001
Sensitivity model + GCS	Hemorrhagic stroke vs. ischemic stroke	2.02 (1.46–2.79)	<0.001
Sensitivity model + GCS	Pneumonia	5.25 (4.05–6.80)	<0.001
Sensitivity model + GCS	Urinary tract infection	1.04 (0.74–1.46)	0.816
Sensitivity model + GCS	Pressure sore/pressure sore infection	0.87 (0.54–1.41)	0.572
Sensitivity model + GCS	Sepsis-related coding	10.70 (4.89–23.40)	<0.001
Sensitivity model + GCS	Initial GCS, per point	0.78 (0.75–0.81)	<0.001
Sensitivity model + GCS	Age, per year	1.02 (1.01–1.04)	<0.001
Sensitivity model + GCS	Atrial fibrillation	1.55 (1.17–2.06)	0.002
Sensitivity model + GCS	Diabetes mellitus	1.43 (1.06–1.92)	0.017
Sensitivity model + GCS	Renal disease	1.67 (1.19–2.36)	0.003

Note: Model performance: primary model N = 5395, AUC = 0.885, McFadden pseudo-R^2^ = 0.340; GCS sensitivity model N = 2971, AUC = 0.920, McFadden pseudo-R^2^ = 0.448. GCS, Glasgow Coma Scale; OR, odds ratio; CI, confidence interval.

**Table 6:** Incremental mortality-related contribution of infectious complications.

Analysis Block	N	Deaths	AUC Reduced	AUC Full	ΔAUC	Mcfadden R^2^ Reduced	McFadden R^2^ Full	ΔR^2^	AIC Reduced	AIC Full	ΔAIC	LR χ^2^	df	LR *p* Value
Primary cohort, no GCS	5395	977	0.826	0.885	+0.058	0.228	0.340	+0.112	3984.8	3421.2	−563.6	571.6	4	<0.001
Sensitivity cohort with GCS	2971	591	0.890	0.920	+0.030	0.377	0.448	+0.072	1896.3	1691.5	−204.8	212.8	4	<0.001

Note: Reduced models were clinical-only models. Full models added individual infectious complications to the corresponding clinical model. In the GCS-available subset, both the reduced and full models included GCS. Adding infectious complications significantly improved model fit and discrimination in both analyses. AIC, Akaike information criterion; AUC, area under the receiver operating characteristic curve; LR, likelihood ratio.

## Data Availability

The data analyzed in this study were derived from routinely collected institutional clinical records. Due to institutional and ethical restrictions, the dataset is not publicly available, but may be made available from the corresponding author on reasonable request and with permission of the hosting institution.

## Supplementary Materials

The following supporting information can be downloaded at: https://www.mdpi.com/article/10.3390/jcm15145562/s1, Table S1: Variable completeness and missingness in the analytical database; Table S2: Model validation and calibration analysis; Table S3: Mortality according to pneumonia category in the COVID sensitivity analysis; Table S4: COVID-related sensitivity analysis: adjusted infectious predictors of in-hospital mortality; Table S5: Comparison between complete-case and excluded observations for the main multivariable models; Table S6: Multicollinearity assessment using variance inflation factors and tolerance values; Figure S1: Calibration curve for the primary no-GCS model; Figure S2: Calibration curve for the GCS-adjusted sensitivity model; Figure S3: Decision curve analysis for the primary no-GCS model; Figure S4: Decision curve analysis for the GCS-adjusted sensitivity model.

## Abbreviations

The following abbreviations are used in this manuscript:

AIC	Akaike information criterion
AUC	Area under the receiver operating characteristic curve
BP	Blood pressure
CI	Confidence interval
COVID	Coronavirus disease
df	Degrees of freedom
GCS	Glasgow Coma Scale
ICU	Intensive care unit
IQR	Interquartile range
LR	Likelihood ratio
OR	Odds ratio
pp	Percentage points
SD	Standard deviation
TIA	Transient ischemic attack
UTI	Urinary tract infection

## References

[1] Busl K.M. (2019). Healthcare-Associated Infections in the Neurocritical Care Unit. Curr. Neurol. Neurosci. Rep..

[2] Lord A.S., Nicholson J., Lewis A. (2019). Infection Prevention in the Neurointensive Care Unit: A Systematic Review. Neurocrit. Care.

[3] Abulhasan Y.B., Rachel S.P., Châtillon-Angle M.O., Alabdulraheem N., Schiller I., Dendukuri N., Angle M.R., Frenette C. (2018). Healthcare-associated infections in the neurological intensive care unit: Results of a 6-year surveillance study at a major tertiary care center. Am. J. Infect. Control.

[4] European Centre for Disease Prevention and Control (2026). Healthcare-associated infections acquired in intensive care units. ECDC. Annual Epidemiological Report for 2022.

[5] Vincent J.L., Sakr Y., Singer M., Martin-Loeches I., Machado F.R., Marshall J.C., Finfer S., Pelosi P., Brazzi L., Aditianingsih D. (2020). Prevalence and Outcomes of Infection Among Patients in Intensive Care Units in 2017. JAMA.

[6] Westendorp W.F., Nederkoorn P.J., Vermeij J.D., Dijkgraaf M.G., van de Beek D. (2011). Post-stroke infection: A systematic review and meta-analysis. BMC Neurol..

[7] Awere-Duodu A., Darkwah S., Osman A.H., Donkor E.S. (2024). A systematic review and meta-analysis show a decreasing prevalence of post-stroke infections. BMC Neurol..

[8] Smith C.J., Kishore A.K., Vail A., Chamorro A., Garau J., Hopkins S.J., Di Napoli M., Kalra L., Langhorne P., Montaner J. (2015). Diagnosis of Stroke-Associated Pneumonia: Recommendations From the Pneumonia in Stroke Consensus Group. Stroke.

[9] Kishore A.K., Jeans A.R., Garau J., Bustamante A., Kalra L., Langhorne P., Chamorro A., Urra X., Katan M., Napoli M.D. (2019). Antibiotic treatment for pneumonia complicating stroke: Recommendations from the pneumonia in stroke consensus (PISCES) group. Eur. Stroke J..

[10] Weng Y., Shen T. (2025). The impact of post-stroke pneumonia on survival and functional outcomes: A systematic review and meta-analysis. Pak. J. Med. Sci..

[11] Poisson S.N., Johnston S.C., Josephson S.A. (2010). Urinary tract infections complicating stroke: Mechanisms, consequences, and possible solutions. Stroke.

[12] Labeau S.O., Afonso E., Benbenishty J., Blackwood B., Boulanger C., Brett S.J., Calvino-Gunther S., Chaboyer W., Coyer F., Deschepper M. (2021). Prevalence, associated factors and outcomes of pressure injuries in adult intensive care unit patients: The DecubICUs study. Intensive Care Med..

[13] Coman A., Pop D., Muresan F., Oprescu F., Fjaagesund S. (2025). Surveillance and Reporting of Hospital-Associated Infections-A Document Analysis of Romanian Healthcare Legislation Evolution over 20 Years. Healthcare.

[14] Mlendea Gălbineanu S.I.A., Kraft A., Falup-Pecurariu C., Melicianu T.G., Nedelcu L.D. (2026). Healthcare-Associated Infections in Deceased Stroke Patients in a Romanian Neurological ICU: A Retrospective Descriptive Study. Microorganisms.

[15] Vincent J.L., Rello J., Marshall J., Silva E., Anzueto A., Martin C.D., Moreno R., Lipman J., Gomersall C., Sakr Y. (2009). International study of the prevalence and outcomes of infection in intensive care units. JAMA.

[16] Collins G.S., Reitsma J.B., Altman D.G., Moons K.G. (2015). Transparent Reporting of a multivariable prediction model for Individual Prognosis or Diagnosis (TRIPOD): The TRIPOD statement. Ann. Intern. Med..

[17] Van Calster B., McLernon D.J., van Smeden M., Wynants L., Steyerberg E.W. (2019). Topic Group ‘Evaluating diagnostic tests and prediction models’ of the STRATOS initiative. Calibration: The Achilles heel of predictive analytics. BMC Med..

[18] Riley R.D., Ensor J., Snell K.I.E., Harrell F.E., Martin G.P., Reitsma J.B., Moons K.G.M., Collins G., van Smeden M. (2020). Calculating the sample size required for developing a clinical prediction model. BMJ.

[19] Badve M.S., Zhou Z., van de Beek D., Anderson C.S., Hackett M.L. (2019). Frequency of post-stroke pneumonia: Systematic review and meta-analysis of observational studies. Int. J. Stroke.

[20] Grossmann I., Rodriguez K., Soni M., Joshi P.K., Patel S.C., Shreya D., Zamora D.I., Patel G.S., Sange I. (2021). Stroke and Pneumonia: Mechanisms, Risk Factors, Management, and Prevention. Cureus.

[21] de Jonge J.C., van de Beek D., Lyden P., Brady M.C., Bath P.M., van der Worp H.B. (2022). Temporal Profile of Pneumonia After Stroke. Stroke.

[22] Singer M., Deutschman C.S., Seymour C.W., Shankar-Hari M., Annane D., Bauer M., Bellomo R., Bernard G.R., Chiche J.D., Coopersmith C.M. (2016). The Third International Consensus Definitions for Sepsis and Septic Shock (Sepsis-3). JAMA.

[23] Evans L., Rhodes A., Alhazzani W., Antonelli M., Coopersmith C.M., French C., Machado F.R., Mcintyre L., Ostermann M., Prescott H.C. (2021). Surviving sepsis campaign: International guidelines for management of sepsis and septic shock 2021. Intensive Care Med..

[24] Blot S., Ruppé E., Harbarth S., Asehnoune K., Poulakou G., Luyt C.E., Rello J., Klompas M., Depuydt P., Eckmann C. (2022). Healthcare-associated infections in adult intensive care unit patients: Changes in epidemiology, diagnosis, prevention and contributions of new technologies. Intensive Crit. Care Nurs..

[25] Teasdale G., Jennett B. (1974). Assessment of coma and impaired consciousness. A practical scale. Lancet.

[26] Steyerberg E.W., Harrell F.E., Borsboom G.J., Eijkemans M.J., Vergouwe Y., Habbema J.D. (2001). Internal validation of predictive models: Efficiency of some procedures for logistic regression analysis. J. Clin. Epidemiol..

[27] Wolff R.F., Moons K.G.M., Riley R.D., Whiting P.F., Westwood M., Collins G.S., Reitsma J.B., Kleijnen J., Mallett S. (2019). PROBAST Group†. PROBAST: A Tool to Assess the Risk of Bias and Applicability of Prediction Model Studies. Ann. Intern. Med..

[28] Rosenthal V.D., Myatra S.N., Divatia J.V., Biswas S., Shrivastava A., Al-Ruzzieh M.A., Ayaad O., Bat-Erdene A., Bat-Erdene I., Narankhuu B. (2022). The impact of COVID-19 on health care-associated infections in intensive care units in low- and middle-income countries: International Nosocomial Infection Control Consortium (INICC) findings. Int. J. Infect. Dis..

[29] Weiner-Lastinger L.M., Pattabiraman V., Konnor R.Y., Patel P.R., Wong E., Xu S.Y., Smith B., Edwards J.R., Dudeck M.A. (2022). The impact of coronavirus disease 2019 (COVID-19) on healthcare-associated infections in 2020: A summary of data reported to the National Healthcare Safety Network. Infect. Control Hosp. Epidemiol..

[30] Tiu C., Terecoasă E.O., Tuță S., Bălașa R., Simu M., Sabău M., Stan A., Radu R.A., Tiu V., Cășaru B. (2023). Quality of acute stroke care in Romania: Achievements and gaps between 2017 and 2022. Eur. Stroke J..

[31] Shintani A.K., Girard T.D., Eden S.K., Arbogast P.G., Moons K.G., Ely E.W. (2009). Immortal time bias in critical care research: Application of time-varying Cox regression for observational cohort studies. Crit. Care Med..

