# Supplementary material for: Hospital-Course Infectious Complications Associated with In-Hospital Mortality in a Neurological Intensive Care Cohort: A Six-Year Retrospective Study
Source: J Clin Med. 2026 Jul 15;15(14):5562. doi: 10.3390/jcm15145562 (PMC13413336; doi:10.3390/jcm15145562)
Supplement: Supplementary file 1 [file jcm-15-05562-s001.zip › jcm-4408548-supplementary.pdf]

Supplementary material

Supplementary Table S1. Variable completeness and missingness in the analytical database

Cohort: neurological ICU admissions, 2020-2025. Unit of analysis: ICU admission episode. Total analytical cohort: N=5,509 episodes.

Variable	Total N	Complete n	Complete %	Missing/blank n	Missing/blank %
clinical_value_quality_flag	5,509	55	1.0	5,454	99.0
sepsis_detail_original	5,509	85	1.5	5,424	98.5
date_quality_flag	5,509	113	2.1	5,396	97.9
pressure_ulcer_detail_original	5,509	202	3.7	5,307	96.3
map_initial	5,509	513	9.3	4,996	90.7
urinary_infection_detail_original	5,509	822	14.9	4,687	85.1
survival_days_recorded	5,509	990	18.0	4,519	82.0
death_date	5,509	999	18.1	4,510	81.9
survival_days_calculated	5,509	998	18.1	4,511	81.9
pneumonia_detail_original	5,509	1,222	22.2	4,287	77.8
ct_initial_documented	5,509	1,257	22.8	4,252	77.2
gcs_initial	5,509	3,007	54.6	2,502	45.4
source_nr_crt	5,509	4,531	82.2	978	17.8
discharge_date	5,509	4,666	84.7	843	15.3
diastolic_bp_initial	5,509	4,968	90.2	541	9.8
systolic_bp_initial	5,509	4,991	90.6	518	9.4
age	5,509	5,434	98.6	75	1.4
sex_male	5,509	5,484	99.5	25	0.5
obesity	5,509	5,506	99.9	3	0.1
length_of_stay_days_calculated	5,509	5,503	99.9	6	0.1
pressure_ulcer_infection	5,509	5,505	99.9	4	0.1
hemorrhagic_stroke	5,509	5,509	100.0	0	0.0
transient_ischemic_attack	5,509	5,509	100.0	0	0.0
ischemic_thrombolysis_mentioned	5,509	5,509	100.0	0	0.0
ischemic_stroke	5,509	5,509	100.0	0	0.0
sex	5,509	5,509	100.0	0	0.0
admission_or_diagnosis_date_flag	5,509	5,509	100.0	0	0.0
death	5,509	5,509	100.0	0	0.0
death_date_flag	5,509	5,509	100.0	0	0.0
discharge_date_flag	5,509	5,509	100.0	0	0.0
source_year	5,509	5,509	100.0	0	0.0
source_row_number	5,509	5,509	100.0	0	0.0
admission_or_diagnosis_date	5,509	5,507	>99.9	2	<0.1
study_id	5,509	5,509	100.0	0	0.0
pneumonia	5,509	5,508	>99.9	1	<0.1
covid_pneumonia	5,509	5,509	100.0	0	0.0
stroke_sequelae	5,509	5,508	>99.9	1	<0.1
diabetes	5,509	5,508	>99.9	1	<0.1
atrial_fibrillation	5,509	5,508	>99.9	1	<0.1
ischemic_heart_disease_mi	5,509	5,508	>99.9	1	<0.1
hypertension	5,509	5,508	>99.9	1	<0.1
any_infection	5,509	5,509	100.0	0	0.0
sepsis_related	5,509	5,509	100.0	0	0.0
stroke_type_main	5,509	5,509	100.0	0	0.0
urinary_infection	5,509	5,508	>99.9	1	<0.1
anticoagulation	5,509	5,509	100.0	0	0.0
parkinson_mentioned	5,509	5,509	100.0	0	0.0
previous_stroke	5,509	5,509	100.0	0	0.0
coagulopathy	5,509	5,509	100.0	0	0.0
hepatic_disease	5,509	5,508	>99.9	1	<0.1
malignancy	5,509	5,509	100.0	0	0.0
renal_disease	5,509	5,509	100.0	0	0.0
potential_identical_duplicate	5,509	5,509	100.0	0	0.0

Note. The variables ending in _detail_original represent original source-detail or free-text documentation fields and should not be interpreted as missingness of the derived binary infection variables used in the main analyses. Their availability largely reflects the presence of additional infection-specific documentation among positive cases. The binary variables used for analysis were pneumonia, urinary tract infection, pressure sore or pressure sore-related infection, sepsis-related coding, COVID-related pneumonia, and any infectious complication.

Supplementary Table S2. Model validation and calibration analysis

Model	N	Deaths	Predictors	AUC	Brier score	Log loss	Calibration intercept	Calibration slope	HL chi2	HL p value	Bootstrap-corrected AUC	Bootstrap-corrected Brier	Bootstrap-corrected slope	Successful bootstrap resamples
Primary model: clinical + individual infections, no GCS	5395	977	26	0.8846	0.0984	0.3121	0	1	33.486	0.0001	0.8795	0.1005	0.9670	100
Sensitivity model: clinical + individual infections + GCS	2971	591	27	0.9198	0.0841	0.2753	0	1	14.287	0.0746	0.9147	0.0873	0.9505	97

Note. The statistically significant Hosmer-Lemeshow test in the primary model should be interpreted cautiously because of the large sample size. Bootstrap-corrected discrimination and calibration remained acceptable, supporting internal model robustness. HL, Hosmer-Lemeshow.

Supplementary Table S3. Mortality according to pneumonia category in the COVID sensitivity analysis

Pneumonia category	N	Deaths	Mortality rate
No pneumonia	4287	396	9.2%
Non-COVID pneumonia	1089	551	50.6%
COVID-related pneumonia	133	52	39.1%

Note. Pneumonia categories are mutually exclusive for this sensitivity analysis.

Supplementary Table S4. COVID-related sensitivity analysis: adjusted infectious predictors of in-hospital mortality

Model	Predictor	Adjusted OR (95% CI)	p value
Primary standard model, no GCS	Pneumonia	6.82 (5.70-8.18)	<0.001
Primary standard model, no GCS	Urinary tract infection	0.92 (0.73-1.16)	0.487
Primary standard model, no GCS	Pressure sore / pressure sore infection	1.17 (0.81-1.68)	0.407
Primary standard model, no GCS	Sepsis-related coding	12.40 (6.53-23.54)	<0.001
COVID-excluded model, no GCS	Pneumonia	7.43 (6.15-8.98)	<0.001
COVID-excluded model, no GCS	Urinary tract infection	0.90 (0.71-1.14)	0.387
COVID-excluded model, no GCS	Pressure sore / pressure sore infection	1.22 (0.84-1.78)	0.298
COVID-excluded model, no GCS	Sepsis-related coding	12.33 (6.43-23.65)	<0.001
Pneumonia split model, no GCS	Non-COVID pneumonia	7.49 (6.20-9.04)	<0.001
Pneumonia split model, no GCS	COVID-related pneumonia	3.43 (2.25-5.24)	<0.001
Pneumonia split model, no GCS	Urinary tract infection	0.91 (0.72-1.15)	0.426
Pneumonia split model, no GCS	Pressure sore / pressure sore infection	1.16 (0.81-1.67)	0.424
Pneumonia split model, no GCS	Sepsis-related coding	13.03 (6.87-24.71)	<0.001
COVID-excluded model + GCS	Pneumonia	5.66 (4.31-7.42)	<0.001
COVID-excluded model + GCS	Urinary tract infection	0.99 (0.70-1.40)	0.968
COVID-excluded model + GCS	Pressure sore / pressure sore infection	0.95 (0.58-1.56)	0.849
COVID-excluded model + GCS	Sepsis-related coding	10.47 (4.75-23.05)	<0.001
Pneumonia split model + GCS	Non-COVID pneumonia	5.67 (4.33-7.44)	<0.001
Pneumonia split model + GCS	COVID-related pneumonia	2.57 (1.28-5.16)	0.0077
Pneumonia split model + GCS	Urinary tract infection	1.06 (0.75-1.48)	0.754
Pneumonia split model + GCS	Pressure sore / pressure sore infection	0.89 (0.55-1.43)	0.624
Pneumonia split model + GCS	Sepsis-related coding	11.38 (5.23-24.74)	<0.001

Note. The association between pneumonia and in-hospital mortality was not driven exclusively by COVID-related pneumonia. Non-COVID pneumonia remained a strong independent predictor after exclusion of COVID-related pneumonia and in models adjusted for GCS. OR, odds ratio; CI, confidence interval; GCS, Glasgow Coma Scale.

Supplementary Table S5. Comparison between complete-case and excluded observations for the main multivariable models

Variable	Primary no-GCS model included, N=5,395	Primary no-GCS model excluded, N=114	GCS-adjusted model included, N=2,971	GCS-adjusted model excluded, N=2,538
In-hospital mortality, n (%)	977 (18.1%)	22 (19.3%)	591 (19.9%)	408 (16.1%)

Variable	Primary no-GCS model included, N=5,395	Primary no-GCS model excluded, N=114	GCS-adjusted model included, N=2,971	GCS-adjusted model excluded, N=2,538
Age, years, median (IQR)	71.0 (62.0–80.0)	71.0 (65.0–81.0)	71.0 (62.0–80.0)	71.0 (62.0–80.0)
Male sex, n (%)	2903 (53.8%)	38 (42.7%)	1606 (54.1%)	1335 (53.1%)
Any infectious complication, n (%)	1865 (34.6%)	46 (40.4%)	1049 (35.3%)	862 (34.0%)
Pneumonia, n (%)	1196 (22.2%)	26 (23.0%)	686 (23.1%)	536 (21.1%)
Urinary tract infection, n (%)	794 (14.7%)	28 (24.8%)	441 (14.8%)	381 (15.0%)
Pressure sore / pressure sore infection, n (%)	196 (3.6%)	6 (5.5%)	124 (4.2%)	78 (3.1%)
Sepsis-related coding, n (%)	81 (1.5%)	4 (3.5%)	60 (2.0%)	25 (1.0%)
Hemorrhagic stroke, n (%)	694 (12.9%)	11 (9.6%)	414 (13.9%)	291 (11.5%)
Renal disease, n (%)	631 (11.7%)	12 (10.5%)	346 (11.6%)	297 (11.7%)
Hepatic disease, n (%)	471 (8.7%)	13 (11.5%)	195 (6.6%)	289 (11.4%)

Note. The primary no-GCS model excluded only 114 admissions because of incomplete covariate data, with similar crude mortality among included and excluded observations. The GCS-adjusted model excluded a larger number of observations, primarily because initial GCS was not available for the full cohort; therefore, this model was interpreted as a complete-data severity-adjusted sensitivity analysis.

Supplementary Table S6. Multicollinearity assessment using variance inflation factors and tolerance values

Variable	VIF	Tolerance
**Primary no-GCS model**		
Year 2024 vs 2020	2.157	0.464
Year 2022 vs 2020	2.14	0.467
Year 2023 vs 2020	2.064	0.484
Year 2021 vs 2020	1.805	0.554
Year 2025 vs 2020	1.666	0.6
Previous stroke	1.316	0.76
Stroke sequelae vs ischemic stroke	1.3	0.769
Age	1.295	0.772
Coagulopathy	1.247	0.802
Multiple stroke categories vs ischemic stroke	1.24	0.806
Anticoagulation	1.222	0.818
Obesity	1.194	0.838
Atrial fibrillation	1.168	0.856
Pneumonia	1.158	0.863
Hemorrhagic stroke vs ischemic stroke	1.157	0.864
Hypertension	1.119	0.894
Ischemic heart disease / prior MI	1.114	0.898
TIA vs ischemic stroke	1.108	0.902
Male sex	1.094	0.914
Urinary tract infection	1.092	0.916
Hepatic disease	1.066	0.938
Renal disease	1.057	0.946
Pressure sore / pressure sore infection	1.056	0.947
Diabetes mellitus	1.036	0.965
Sepsis-related coding	1.034	0.967
Malignancy	1.018	0.983
**GCS-adjusted sensitivity model**		
Year 2024 vs 2020	2.364	0.423
Year 2025 vs 2020	1.985	0.504
Year 2023 vs 2020	1.807	0.553
Year 2022 vs 2020	1.550	0.645
Initial GCS	1.320	0.758
Age	1.301	0.769
Stroke sequelae vs ischemic stroke	1.289	0.776
Multiple stroke categories vs ischemic stroke	1.286	0.777
Previous stroke	1.263	0.792
Year 2021 vs 2020	1.249	0.801
Coagulopathy	1.244	0.804
Pneumonia	1.241	0.806
Anticoagulation	1.224	0.817
Hemorrhagic stroke vs ischemic stroke	1.220	0.820
Obesity	1.207	0.828
Atrial fibrillation	1.183	0.846
Hypertension	1.125	0.889
Ischemic heart disease / prior MI	1.118	0.895
TIA vs ischemic stroke	1.111	0.900
Male sex	1.106	0.905
Urinary tract infection	1.103	0.907
Hepatic disease	1.066	0.938
Pressure sore / pressure sore infection	1.061	0.942
Renal disease	1.060	0.944
Sepsis-related coding	1.052	0.951
Diabetes mellitus	1.051	0.952
Malignancy	1.019	0.982

Note. Variance inflation factors and tolerance values were calculated for the primary no-GCS model and the GCS-adjusted sensitivity model. No relevant multicollinearity was detected, as all VIF values were below 5 and all tolerance values were above 0.20.

Supplementary Figure S1. Calibration curve for the primary no-GCS model.

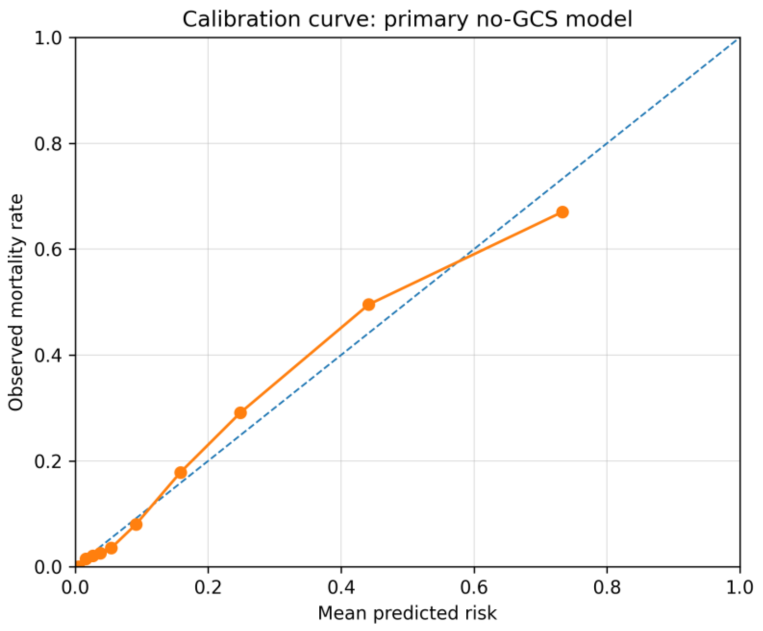

Note. The curve compares mean predicted risk with observed in-hospital mortality by deciles of predicted risk. The diagonal reference line indicates perfect calibration.

Supplementary Figure S2. Calibration curve for the GCS-adjusted sensitivity model

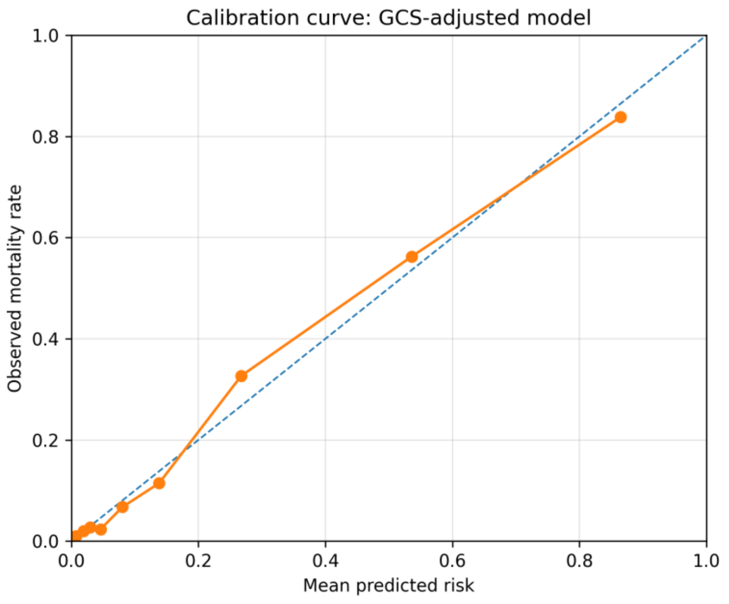

Note. The curve compares mean predicted risk with observed in-hospital mortality by deciles of predicted risk. The diagonal reference line indicates perfect calibration.

Supplementary Figure S3. Decision curve analysis for the primary no-GCS model

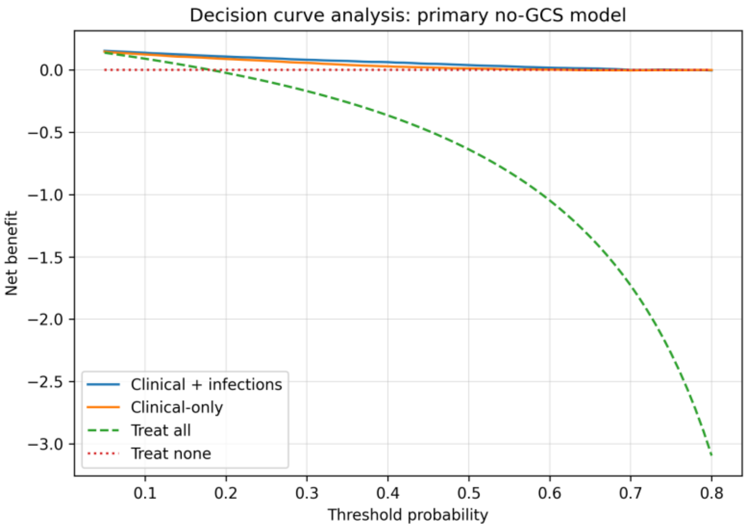

Note. Net benefit is shown across threshold probabilities for the clinical-only model, the clinical-plus-infections model, the treat-all strategy, and the treat-none strategy.

Supplementary Figure S4. Decision curve analysis for the GCS-adjusted sensitivity model

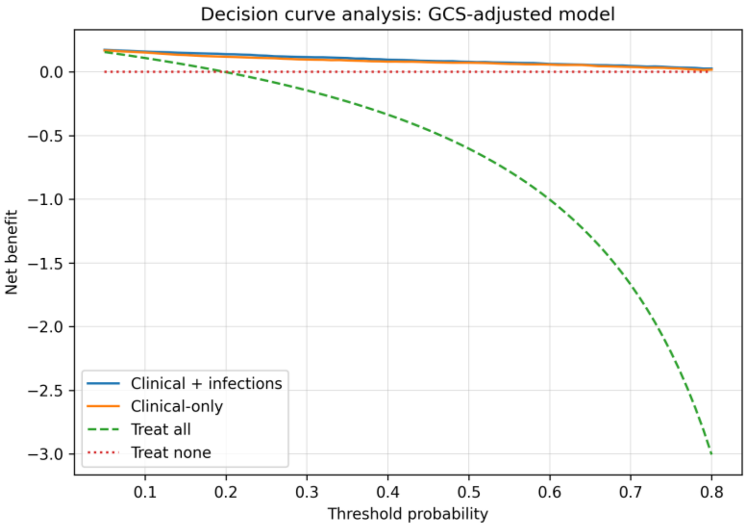

Note. Net benefit is shown across threshold probabilities for the clinical-only model, the clinical-plus-infections model, the treat-all strategy, and the treat-none strategy.
